# Circulating tumor DNA and Response Evaluation Criteria In Solid Tumors: ctDNA-RECIST proof-of-concept in HER2-positive metastatic breast cancer

**DOI:** 10.1186/s13046-025-03605-2

**Published:** 2026-01-20

**Authors:** Alessandra Fabi, Elena Giordani, Elena Ricciardi, Grazia Arpino, Matteo Allegretti, Gianluigi Ferretti, Claudia Omarini, Alberto Zambelli, Chiara Mandoj, Andrea Botticelli, Emilio Bria, Stefania Gori, Luisa Carbognin, Ida Paris, Giovanni Scambia, Francesco Cognetti, Diana Giannarelli, Patrizio Giacomini

**Affiliations:** 1 Precision Medicine Unit in Senology, Fondazione Policlinico Universitario Agostino Gemelli IRCCS, Largo Agostino Gemelli, 8, Rome, 00168 Italy; 2 Translational Oncology Research, IRCCS Regina Elena National Cancer Institute, Rome, Italy; 3 Oncology Division, Department of Clinical Medicine and Surgery, University Federico II, Naples, Italy; 4 Division of Medical Oncology 1, IRCCS Regina Elena National Cancer Institute, Rome, Italy; 5 Division of Medical Oncology, Department of Oncology and Hematology, University Hospital of Modena, Modena, Italy; 6 Oncology Unit, ASST Papa Giovanni XXIII, Bergamo, Italy; 7 Oncology, Department of Medicine and Surgery, University of Milano-Bicocca, Milan, Italy; 8 Clinical Pathology Unit and Cancer Biobank, IRCCS Regina Elena National Cancer Institute, Rome, Italy; 9 Department of Radiological, Oncological and Pathological Science, Sapienza University of Rome, Rome, Italy; 10 Università Cattolica del Sacro Cuore, Rome, Fondazione Policlinico Universitario Agostino Gemelli IRCCS, Rome, Italy; 11 Ospedale Isola Tiberina – Gemelli Isola, Rome, Italy; 12 Medical Oncology, IRCCS-Sacro Cuore Don Calabria Hospital, Negrar Di Valpolicella, Verona, Italy; 13 Gynaecological Oncology, Fondazione Policlinico Universitario Agostino Gemelli IRCCS, Rome, Italy; 14 Gynaecologic Oncology Unit, Department of Woman and Child Health and Public Health, Fondazione Policlinico Universitario Agostino Gemelli IRCCS, Rome, Italy; 15 Section of Obstetrics and Gynaecology, University Department of Life Sciences and Public Health, Università Cattolica del Sacro Cuore, Rome, Italy; 16 Facility of Epidemiology and Biostatistics, Fondazione Policlinico Universitario Agostino Gemelli IRCCS, Rome, Italy; 17 European Liquid Biopsy Society (ELBS), Hamburg, Germany

**Keywords:** CtDNA, RECIST 1.1, CtDNA-RECIST, HER2-positive Breast Cancer, T-DM1, Objective response evaluation

## Abstract

**Background:**

Response Evaluation Criteria In Solid Tumors (RECIST 1.1) and circulating tumor DNA (ctDNA) recapitulate and anticipate response to treatment, respectively. However, ctDNA-RECIST (cRECIST) and ctDNA-guided End of Treatment (cEoT) are not applied routinely.

**Methods:**

To provide proof-of-concept for RECIST1.1/cRECIST integration, HER2-positive metastatic breast cancer patients (*n* = 50) were enrolled in the multi-center prospective GIM21 study to receive Trastuzumab-emtansine (T-DM1). CT scans (113 tumor lesions) were longitudinally assessed for classical Objective Responses (ORs: progressive disease/stable disease/partial response/complete response; PD/SD/PR/CR) applying default RECIST 1.1 cut-offs (SD/PD ≥ 20%; SD/PR ≤ 30%). Likewise, bespoke NGS/dPCR (78 genomic alterations; 466 time points) were converted into ctDNA-Objective Responses (cORs: cPD/cSD/cPR/cCR) exploring wide cPD/cSD/cCR cut-off ranges, both default (RECIST 1.1-like) and alternative.

**Results:**

Whichever the cut-off, cORs were much deeper than ORs, leading to RECIST 1.1/cRECIST divergence in 27 cPD-positive patients. Moreover, due to complex ctDNA trajectories (multiple successive ctDNA increases/decreases, termed ctDNA waving), cPD (the earliest ctDNA increase) correlated with outcome in broad patient subsets but not individual patients. To deconvolute ctDNA waving, cPD was combined with three-point ctDNA *Tr*ends (*Tr*), resulting in a personalized cEoT clinical algorithm that, once retrofitted to the 27 cPD-positive patient dataset, aligned with PFS much better than cPD (cEoT/PFS vs cPD/PFS linear regression: R^2^ = 0.85 vs 0.35).

**Conclusions:**

Even in difficult ctDNA scenarios, the cEoT algorithm may help to: (a) predict treatment efficacy during drug development, (b) adaptively randomize for patient-specific, timely treatment switch in clinical trials, and (c) prevent premature treatment withdrawal in long-responders. Future randomized studies are warranted for cRECIST/RECIST 1.1 integration/personalization in different tumors/settings.

**Trial registration:**

NCT05735392.

**Supplementary Information:**

The online version contains supplementary material available at 10.1186/s13046-025-03605-2.

## Background

Response Evaluation Criteria In Solid Tumors (RECIST) 1.1 assess clinical objective response (OR) to treatment in metastatic cancer based on dimensional changes of tumor lesions measured by medical imaging. The RECIST 1.1 scale, metrics and conventions guide oncology practice, define measurable endpoints in clinical trials, and ultimately provide an objective framework for the regulatory approval of new drugs [1]. However, the limitations of a purely anatomical assessment of response to treatment are widely recognized, and additional RECIST criteria have been proposed to evaluate Positron Emission Tomography (PET) and immune Response (PERCIST and iRECIST/imRECIST) [2, 3]. Alternative and additional criteria apply to lymphomas [4] and organ-specific assessment, such as brain metastases [5].

The potential advantages to integrate circulating tumor DNA (ctDNA) into RECIST 1.1 (and possibly other objective response scales) have been outlined [6–9]. The EORTC RECIST working group (https://recist.eortc.org) and the European Liquid Biopsy Society (www.ELBS.eu) are at work to provide guidelines and recommendations for ctDNA-RECIST (cRECIST hitherto). However, publicly available ctDNA datasets are presently much smaller than the medical imaging datasets interrogated during successive RECIST refinements. Moreover, although changes in ctDNA levels may effectively stratify patients into good and poor responders [10–20], no prospective clinical studies have been reported, to our knowledge, to estimate ctDNA-adjusted risk of clinical progression in individual patients.

Herein, we report on a multi-center prospective study, designed by the Gruppo Italiano Mammella (GIM) and called GIM21/LiqERBcept (NCT05735392). The primary aim of GIM21 was to combine/integrate ctDNA and medical imaging (RECIST 1.1) in HER2-positive, metastatic breast cancer patients receiving the first-in-class Antibody–Drug Conjugate (ADC) Trastuzumab emtansine (T-DM1) as standard of care second-line treatment at the time of study design [21]. Despite T-DM1 has now been superseded in this indication by an irinotecan-based Trastuzumab conjugate (Trastuzumab deruxtecan; T-DXd) [22], T-DM1 treatment remains methodologically appealing, since loss of HER2 amplification coincides with the expansion of new genomic alterations in blood [23]. This oncogenic switch, captured by ctDNA, may be instrumental to align objective tumor responses, contraction/expansion of ctDNA variants, T-DM1 efficacy, and ultimately outcome, e.g. to model a self-consistent cRECIST/RECIST 1.1 framework.

In the GIM21 study, we have prospectively investigated for the first time how changes in ctDNA levels (a continuous variable) may be translated into an objective discontinuous cRECIST scale. Similar to the 4 classical RECIST 1.1 Objective Responses (ORs), e g. Progressive Disease, Stable Disease, Partial Response and Complete Response (PD, SD, PR and CR), cRECIST was postulated to feature 4 ctDNA-ORs (cORs), e.g. cPD, cSD, cPR and cCR. cRECIST is proposed herein to be robust and intuitive enough to stratify patients by disease outcome and, with caveats and additions, also to guide personalized decisions in clinical trials and everyday clinical practice.

## Methods

### Patients

GIM21 is an open-label, observational (blood drawing implemented as an additional procedure), multicenter, single-arm, prospective study (NCT05735392) conducted between July 2018 (first patient in) and December 2022 (last blood drawing). It recruited consecutive adult patients with a documented diagnosis of HER2-positive metastatic BC, as per ImmunoHistoChemistry score (IHC 3 +, or IHC 2 + and HER2 amplification ratio ≥ 2.0). Eligible patients had no more than one line of previous anti-HER2 treatment for advanced disease (Trastuzumab, either alone or combined with Pertuzumab plus taxane). For additional inclusion and exclusion criteria, age, demographics and clinical information see Supplemental Methods and Table S1. T-DM1 was administered at the standard dose of 3.6 mg/kg Q21 days until disease progression or development of unmanageable toxic effects (see Supplemental Methods). All GIM21 patients were monitored until clinical progression during the GIM21 study. After switch to subsequent therapy regimens, patients were monitored until September 1, 2024.

### Sample size and pre-planned analysis

The GIM21 sample size was calculated to detect significant differences in the ranks (Wilcoxon test) between the time elapsed until clinical progression (PFS) and the time elapsed until the first detectable ctDNA increase. In a similar previous study on 20 patients [23], 16 (80%) were ctDNA-positive, and 11 (55%) displayed a measurable ctDNA increase. On this basis, a sample size of 45 GIM21 patients was expected to result in 36 (95% CI: 29 to 41) ctDNA-positives and 25 patients undergoing a measurable ctDNA increase. This would allow to assess with a power of 80% differences in ranks corresponding to an effect size of 0.6 at a significance level of 5%.

### Clinical samples and medical imaging

**Fig. 1:**
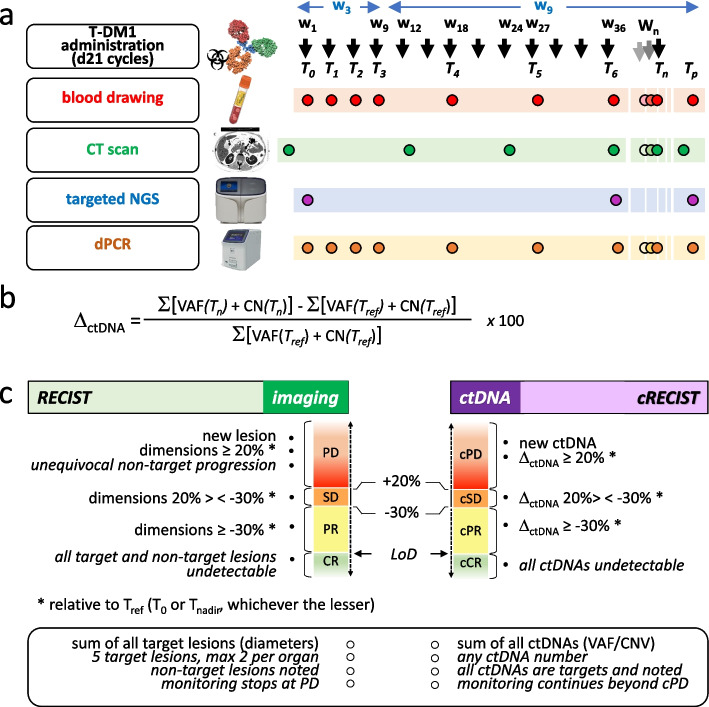
Sample and data collection, cRECIST metrics and notations. **a** Timelines of: T-DM1 administration (top), blood collection (red), medical imaging (green), and testing (NGS and dPCR, blue and yellow). T: time intervals; w: weeks. **b** Formula to calculate Δ_ctDNA_, e.g. % change of all measured target ctDNAs between two points (T_n_ vs T_ref_), whereby T_n_ is the test point in a multipoint series, and T_ref_ is typically either T_0_ or T_nadir_. **c** Mirroring of classical RECIST 1.1 into ctDNA-RECIST (cRECIST). The same cut-off values (≥ 20%, ≤ 30%) are applied by default to classify % dimensional changes in target lesions and ctDNA abundance (Δ_ctDNA_), resulting in 4 possible objective responses (ORs/cORs: PD/cPD, SD/cSD, PR/cPR, and CR/cCR). Few differences are acknowledged between RECIST 1.1 and cRECIST (boxed)

To capture early ctDNA changes, commonly observed during T-DM1 treatment [23], blood was obtained right before treatment (baseline, T_0_), and then at 3 progressively longer intervals: 3 weeks (T_1_ to T_3_), 9 weeks (T_4_ to T_6_), and 12 weeks until progression (T_7_ to T_p_). CT scans were instead obtained as per standard of care: at progression from previous treatment, and then every 12 weeks. This resulted in a ‘staggered’ (w3/w9/w12 vs w12) collection scheme until T_6_, followed by ctDNA/CT scan alignment (Fig. 1a, compare green and red timelines). Blood was drawn immediately before (minutes) T-DM1 infusion at all time points except T_p_, that coincided with the last GIM21 visit (end of treatment), on average 7 days after the final CT scan documenting clinical progression. Technical details on blood drawing and pre-analytical processing are provided in supplementals and elsewhere [23].

### NGS and dPCR

All techniques were previously described [23]. A targeted NGS panel (Oncomine Pan-Cancer) was used detecting: (a) SNVs and INDELs from 52 major cancer drivers and tumor suppressor genes, (b) DNA copy number variations (CNV) from 12 genes, and (c) rearrangements from 12 genes. Alterations were called by NGS at 3 selected time points (T_0_, T_6_ and T_p_; Fig. 1a, blue timeline), except when progression occurred at or before T_6_, which resulted in two NGS testing points only (T_0_ and T_p_). Alterations captured by NGS were systematically monitored (T_0_ to T_p_; Fig. 1a, orange timeline) by bespoke digital PCR (dPCR) assays, either custom-designed or commercially obtained. Each dPCR assay was pre-tested on serial dilutions of tumor tissue DNAs (tDNAs) carrying (n ≥ 2) or lacking (n = 1) the alteration being assessed, and was accepted if its Limit of Detection (LoD) was at 0.01% VAF or better. All reagents, equipment, and online NGS/dPCR bioinformatic/interpretation tools were from ThermoFisher Scientific.

### RECIST 1.1 and ctDNA-RECIST (cRECIST)

As per RECIST 1.1 [1], % change in tumor size is routinely expressed by a single unit, e.g. the sum of diameters of target lesions relative to the lowest measurement on study. To similarly evaluate changes in the amounts of all ctDNA species by a single unit, SNV and CNV measurements were rounded to two decimal places and made commensurable by expressing them as absolute values. For instance, a VAF of 1.101% and a CNV of 2.229 were made equal to 1.10 and 2.23. These absolute values were summed (all alterations present at a given time point), and overall % changes in ctDNA abundance were expressed relative to a reference time point (T_n_ vs T_ref_), using Δ_ctDNA_ as the main unit of measure (Fig. 1b). In this calculation, T_n_ is any time point, and T_ref_ is either T_0_ (baseline) or T_nadir_ (lowest ctDNA measurement) whichever the lesser. Δ_ctDNA_ values were computed taking into account results of bespoke dPCR assays measuring 47 SNVs and 3 CNVs, that were systematically performed at all time points. Since NGS testing was sporadic (compare blue and yellow timelines in Fig. 1), it was considered only when dPCR assays could not be designed (4 CNVs). For details see Supplemental Methods. To turn Δ_ctDNA_ into a discontinuous cRECIST scale, the 4 possible objective RECIST 1.1 responses (ORs) were mirrored into 4 corresponding ctDNA objective responses (cORs), e.g. PD/cPD, SD/cSD, PR/cPR and CR/cCR. The same cut-offs defining the upper and lower limits of SD (≥ 20% and ≤ 30% respectively) were applied by default to cSD (hitherto ‘default cut-offs’; Fig. 1c), but were also the subject of extensive exploratory analysis (see below). PD/cPD and CR/cCR were similarly scored upon detection of a new tumor lesion/new ctDNA species, and loss of all detectable lesions/target ctDNAs by CT scan and dPCR respectively (for dPCR LoD, 0.01%VAF, see above). Two exceptions to RECIST 1.1/cRECIST mirroring are acknowledged: (i) the distinction between target and non-target lesions vs complete equivalence of all ‘target’ ctDNAs; (ii) the upper limit in the number of target lesions (n = 5) in RECIST 1.1 vs no pre-defined limit in target ctDNA species. For symmetry, default monitoring by CT scan and dPCR stopped at PD and cPD respectively.

### Statistics

Results were reported by descriptive statistics and 95% CI. Parametric and non-parametric tests were used to evaluate associations between variables. Progression-free survival (PFS) was calculated as the time between the first T-DM1 administration and either the first evidence of progressive disease or the time of last follow-up. Survival was evaluated by the Kaplan–Meier method. Data were elaborated by GraphPad Prism v10 (RRID:SCR_000306) and IBM SPSS statistics (RRID:SCR_002865).

## Results

### GIM21 study flowchart

**Fig. 2:**
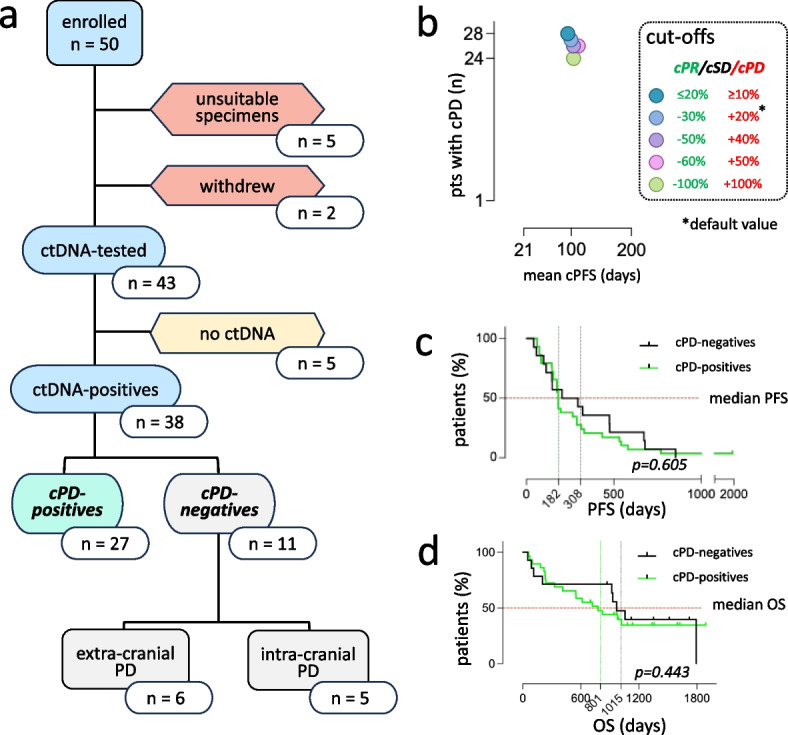
ctDNA-informed GIM21 flowchart. **a** Flowchart summarizing recruitment, attrition, and cPD-based classification of GIM21 patients. **b** Effect of different cOR cut-offs on the number of cPD-positive patients and the mean duration of ctDNA-PFS (cPFS, T_0_ to cPD). cPR: ctDNA Partial Response; cSD: ctDNA Stable Disease; cPD: ctDNA Progressive Disease. **c** and **d** Kaplan–Meier curves: cPD-positive vs cPD-negative patients, default cut-offs. PFS: Progression-Free Survival. OS: Overall Survival. Median PFS and OS are indicated and color-coded for cPD-positives and cPD-negatives. cPD assigned by default cRECIST cut-off values

Five patients were excluded due to either missing blood drawings or poor cfDNA quality. Therefore, reaching the pre-planned sample size of 45 patients required 5 additional patients, resulting in 50 enrolled patients altogether (Fig. 2a). Two patients withdrew from study. Of 43 remaining patients, 5 lacked detectable ctDNA. ctDNA-positives were therefore 38, which was in line with power calculations (36 expected, see Methods). In analogy with the standard RECIST 1.1 cut-off defining PD (≥ 20% dimensional tumor increase), cPD was defined at ≥ 20% Δ_ctDNA_ increase (see Methods and Fig. 1c). Presence of at least one ≥ 20% Δ_ctDNA_ increase sorted the 38 ctDNA-positives into cPD-positives (*n* = 27; 25 expected) and cPD-negatives (*n* = 11). Non-standard cPD cut-offs were also explored, but within the ≥ 10% to ≥ 50% cPD range the number of cPD-positives and the timing of cPD (T_0_ to cPD, e.g. ctDNA-PFS or cPFS) were only marginally affected (Fig. 2b). Therefore, cPD assignment appears to be robust and largely cut-off-independent.

The PD/cPD status was consistent with patient features. For instance, all 27 cPD-positives experienced PD at extra-cranial sites. Conversely, all 5 patients undergoing PD exclusively due to brain metastases were cPD-negative, which is unsurprising given poor ctDNA release from intracranial locations [23, 24]. Finally, the only patient discontinuing T-DM1 due to toxicity did so while in complete objective response, as concordantly assessed by her cPD/PD double-negative status.

Demographics and the clinical pathological features of cPD-positive and cPD-negative patients are separately presented in Table S1. No patient was lost to follow up. At last evaluation, 11/27 cPD-positives and 6/11 cPD-negatives were alive. Despite enrichment in cases with brain metastases, PFS and OS did not significantly differ between cPD-negatives and cPD-positives (Fig. 2c/d). Therefore, detectable cPD did not apparently select for outcome.

### Circulating genomic alterations

Circulating genomic alterations were detected by bespoke NGS/dPCR testing. Supplementary Results provide details about their distribution in patients (oncoprints) and across time points (Fig. S2), their actionable [25] levels (Fig. S3), orthogonal NGS/dPCR validation, selection of dPCR as the standard cRECIST readout, and application of VAF as the optimal metric to measure ctDNA (Fig. S4). Raw ctDNA measurements are provided in an annotated spreadsheet as Table S2.

### Divergent trajectories: tumor lesions vs target ctDNAs

For a detailed longitudinal evaluation of individual trajectories, each of 113 tumor lesions and 78 target ctDNAs was assessed as described in supplemental results (Fig. S4). Compared to tumor lesions, ctDNAs underwent more numerous, frequent, discordant, asynchronous and extreme changes, including occasional direct switch from loss to gain at immediately consecutive time points. In summary, stable disease was rare from the ctDNA standpoint, and evolutionary divergence of ctDNA variants largely exceeded dissociated radiological responses among tumor lesions measured by CT scans. Then, it was of interest to determine whether this translates into a RECIST 1.1/cRECIST divergence.

### RECIST 1.1 vs cRECIST

**Fig. 3:**
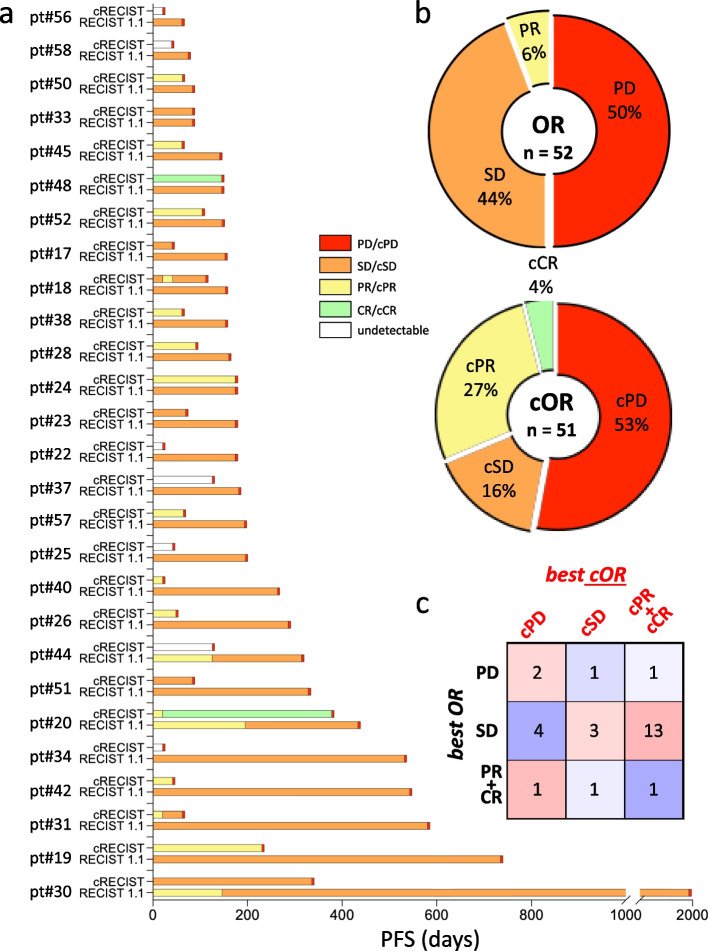
cRECIST vs RECIST 1.1: timelines, frequency and co-occurrence of objective responses. **a** Patient-by-patient bar plots of Objective ctDNA and tumor Responses (cORs top and ORs bottom) as per cRECIST and RECIST 1.1 assessments of the 27 cPD-positive GIM21 patients. Four possible ORs/cORs are noted at the pre-planned time points and color-coded consistently (inset showing PD/cPD, SD/cSD, PR/cPR and CR/cCR), then longitudinally displayed in the timelines until PD and cPD for ORs and cORs respectively. A change in color means change in OR/cOR. Only ctDNA time points in the w3/w9 dataset are considered, e.g. for homogeneous handling of the entire dataset the time-dense w3 dataset was disregarded. **b** Donut charts displaying the observed ORs and cORs by frequency. **c** Pearson’s correlation matrix displaying the number of patients in each of the 9 possible best OR/cOR co-occurrence categories. All elaborations carried out at default cut-off values

To compare RECIST 1.1 and cRECIST, as per GIM21 primary aim, target lesion diameters and Δ_ctDNA_ values were calculated at the pre-planned time points (Fig. 1) from the 27 cPD-positive GIM21 patients. Objective clinical and ctDNA responses (OR and cORs) were longitudinally assigned by application to both tumor lesions and ctDNAs of default RECIST 1.1 cut-offs (SD/PD ≥ 20% and SD/PR ≤ 30%, see Fig. 1). ORs/cORs were color-matched (SD/cSD, PR/cPR, CR/cCR, PD/cPD), and displayed as timelines until PD and cPD respectively (Fig. 3a). As expected, PD and cPD significantly differed in timing (*p* < 0.0001, Wilcoxon test). Comparing bar length between cRECIST and RECIST1.1 revealed that cPD anticipated PD ≥ 21 days (at least one T-DM1 cycle) in 24/27 patients, resulting in much shorter cOR than OR observation periods. Despite this shorter monitoring time, assignments changed more frequently for cORs than ORs (see color patchworks), and the total numbers of recorded responses were similar (*n* = 52 vs 51; donut charts in Fig. 3b). Altogether, cSD was under-represented compared to SD (16%vs 44%), whereas cPR and cCR were over-represented compared to PR and CR (Fig. 3b). In other words, due to divergence between individual tumor lesions and ctDNAs (see Fig. S4), ORs and cORs also diverged.

To further investigate divergence, best Objective Responses (best ORs and best cORs) were derived from the same dataset. For consistency, best OR (classically defined as the greatest recorded tumor shrinkage) was mirrored into best cOR (defined as the deepest Δ_ctDNA_ drop on study). Interestingly, a Pearson’s correlation matrix measuring co-occurrence (PD/cPD, SD/cSD, PR/cPR, and CR/cCR) was significantly (McNemar-Bowker test *p* = 0.007) skewed off-diagonal, particularly toward a dominant best SD/best cPR-cCR combination phenotype seen in 13/27 patients (Fig. 3c). Therefore, ctDNA responses were clearly detectable in almost half of the cPD-positive patients achieving disease stability as their best response. Similar patterns (timelines, pie charts and Pearson’s plots) were seen at upper/lower cSD cut-offs, at least within the ≥ 50% to ≤ 60% range (Figs. S5-S8).

Cut-off-independent cOR and best cOR assignments demonstrated that trajectory divergence between tumor size and ctDNA does not depend on how they are measured, but on an intrinsically deeper response to T-DM1 treatment of ctDNA compared to tumor lesions.

### cRECIST and clinical outcome

**Fig. 4:**
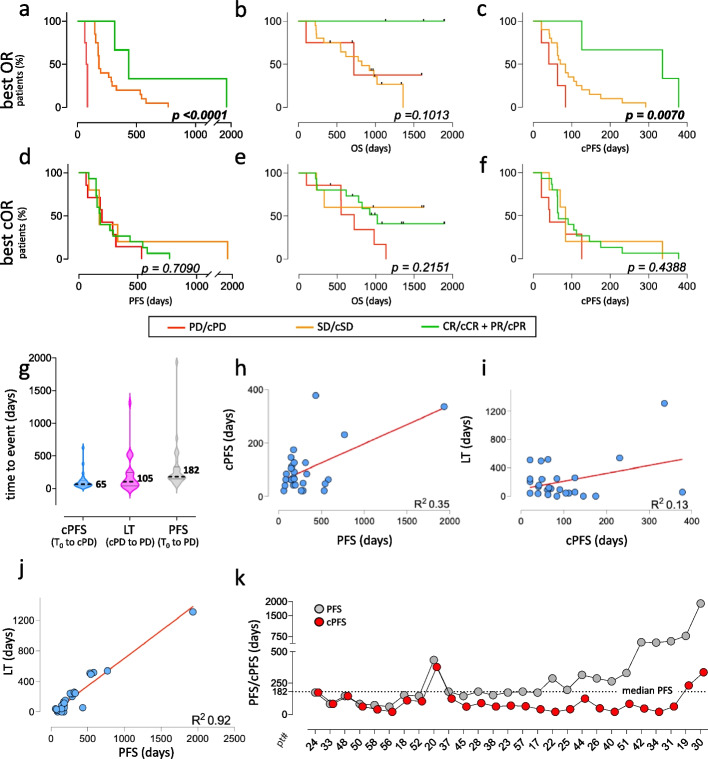
Objective responses and outcome. **a**-**c** Kaplan–Meier curves of 27 cPD-positive patients sorted by best OR (greatest tumor shrinkage). **d**-**f** As above, sorted by best cOR (greatest Δ_ctDNA_ drop). OR: Objective Response. PFS: Progression-Free (PD-free) Survival. cPFS: ctDNA-PFS, eg. cPD-Free Survival. OS: Overall Survival. *p* values are from pooled analysis of PD vs SD vs PR/CR, and cPD vs cSD vs cPR/cCR. **g** Violin plots comparing days [95% CI] for the variables defined in figure, e.g.: ctDNA-PFS (cPFS, 65 [154–47]), Lead Time (LT, 105 [243–42]), and PFS (182 [315–154]). All values calculated for the 27 cPD-positive patients altogether. **h**-**j** linear regression curves (best fit) between any two of LT, PFS, and cPFS, with correlation coefficients. **k** PFS and cPFS plotted by patient number after patient ranking by LT. cORs assigned by default cut-off values in all panels

Next, best objective responses (best ORs and best cORs) were correlated with the clinical outcomes (PFS and OS) of the 27 cPD-positive patients, and with a time-dependent cRECIST variable termed ctDNA-PFS (cPFS). Analogous to PFS, that measures the time elapsed between T_0_ and PD, cPFS was defined as the time elapsed between T_0_ and cPD. Unsurprisingly, tumor shrinkage/best OR correlated with PFS, and a non-significant trend was also noted for OS (Fig. 4a and b). Interestingly, best OR also correlated with cPFS (Fig. 4c). Since best OR correlates with both PFS and cPFS, clinical and ctDNA responses (PFS and cPFS) do correlate, although indirectly. In contrast, and quite disappointing, deepest ctDNA drop/best cOR did not correlate at all with PFS, OS or cPFS (Fig. 4d/f). This was puzzling, since many groups have observed significant correlations between quantitative ctDNA changes and treatment efficacy (see for instance refs [10–20]). Poor correlations in the GIM21 study were not due to cOR/ctDNA being measured on a discontinuous scale, since extensive simulations applying both discontinuous and conventional continuous variables invariably failed to correlate quantitative ctDNA changes with outcome (see supplemental results and Fig. S9).

Then, we disregarded ctDNA amounts and focused on three time-dependent variables: cPFS, Lead Time (LT), and PFS. cPFS and LT define two consecutive treatment periods (T_0_ to cPD, and cPD to PD). PFS measures the two periods altogether (T_0_ to PD). Side-by-side plotting showed that cPFS was shortest and least variable (Fig. 4g). It weakly correlated (by regression analysis) with the PFS of individual patients, and not at all with LT (Fig. 4h and i). In sharp contrast, LT and PFS did correlate (Fig. 4j), resulting in roughly concordant LT/PFS patient ranking, particularly evident when median PFS was exceeded, e.g. in long-term responders (Fig. 4k). Increasing cPD cut-offs resulted in minimal changes in cPFS measurements, and moderately worsened cPFS/best OR linear correlations, minimally if at all affecting LT/PFS correlations (Fig. S10).

Combined, the results in Fig. 4, Fig. S9 and Fig. S10 revealed that cPFS (a time variable) correlates with outcome much better than quantitative ctDNA changes. However, presumably due to its limited variation ranges cPFS remains a poor descriptor of individual responses to T-DM1. Since LT/PFS correlations are tighter and resilient to cut-off changes, outcome may be largely determined post-cPD.

### Combining cRECIST with non-cRECIST outcome indicators

**Fig. 5:**
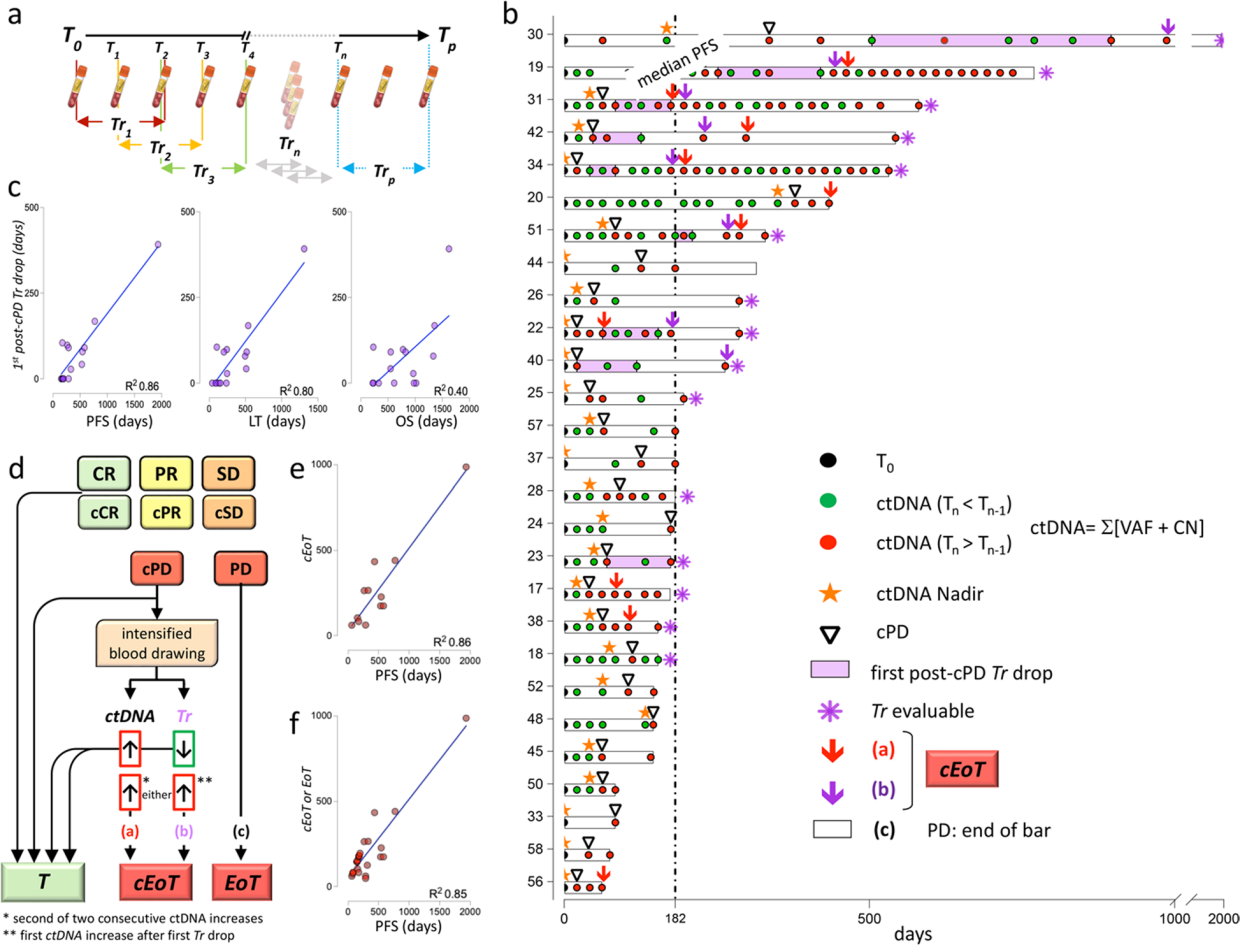
cRECIST, multipoint trends and ctDNA-guided clinical decision algorithm. **a** Schematic Tr calculation: three consecutive time points form a triplet, and triplets overlap by two points. Tr is calculated for each triplet by averaging three consecutive ctDNA measurements, each point as follows: (∑[VAF(Tn) + CN(Tn)]). Series from consecutive overlapping triplets define a ctDNA trend (Tr), either positive or negative. **b** Swimmer plots of the 27 cPD-positive patients displaying clinical/ctDNA ‘placeholders’, listed in the inset as follows. Individual blood drawings (dots) are black at baseline, green when ctDNA decreases and red when ctDNA increases (decrease and increase relative to the previous point). ctDNA_nadir_, cPD, and the duration of the first post-cPD Tr drop are shown. Asterisks identify patients with blood drawing series allowing Tr calculation. The two cEoT metrics (arrows downward) are color-coded and defined in panel d. **c** linear regression plots: duration of the first post-cPD ctDNA decrease (calculated by Tr) vs PFS, LT and OS. **d** GIM21 clinical algorithm and branched decision tree. T: continued treatment, green box. cEoT and EoT, red boxes, treatment discontinued. cEoT/EoT is assigned by (a),(b), or (c), whichever first. **e** and **f** Linear regression plots: time to cEoT vs PFS (*n*=12 patients), and time to either cEoT or EoT whichever first (*n*=27 patients), respectively

It was inferred that it might be inappropriate to describe post-cPD ctDNA changes by comparing series of disconnected paired measurements, each referring to the same reference value (T_n_ vs T_ref_, T_n+1_ vs T_ref_, etc.), as per RECIST 1.1/cRECIST conventions. This might miss individual, informative multipoint ctDNA trends in complex, zig-zagging ctDNA trajectories (see for instance Fig. S3b). Then, the simplest possible multipoint series were considered, e.g. consecutive 3-point series with 2-point overlap (triplets). Δ_ctDNA_ values were measured at each point (T_0_ to T_p_), and then averaged for each triplet, resulting in a triplet *Tr*end value dubbed *Tr* (Fig. 5a). It was observed that none of two consecutive *Tr* values was identical to the second decimal digit, resulting in a simplified scoring system with two alternative trends only (regardless of *Tr* magnitude), e.g. either ctDNA increase (*Tr*_n_ > *Tr*_n-1_) or ctDNA decrease (*Tr*_n_ < *Tr*_n-1_). Disappearance of a previously detected ctDNA, and de novo appearance of a new ctDNA were assimilated to *Tr* decrease and increase respectively. On this basis, swimmer plots were generated displaying the timelines of the 27 cPD-positive patients, all the changes in ctDNA absolute values between consecutive blood drawings, as well as ctDNA_nadir_, cPD, PD, and post-cPD *Tr* decreases (Fig. 5b).

Swimmer plots revealed that: (a) ctDNA_nadir_, cPD and PD invariably occur in this order, e.g. they behave as ‘placeholders’; (b) successive ctDNA and *Tr* increases and decreases, termed ‘ctDNA waving’, are evident in several timeline sections; (c) *Tr* decreases (albeit transient) are preferentially seen in slow-progressors/long-responders (PFS > median PFS; 8/12); (d) PD occurs in 26/27 patients during phases of ctDNA and/or *Tr* increase, and not decrease. Finally, and most interestingly (e), in 15 data-dense patients (asterisks) with at least three consecutive measurable *Tr* values, the duration of the first post-cPD *Tr* decrease significantly correlated with PFS and LT, although not with OS (Fig. 5c). *Tr* was a much better personalized metric than cPFS alone (*Tr* in Fig. 5c vs cPFS in Fig. 4h: R^2^ = 0.86 vs 0.35). This improvement exclusively applied to *Tr* drops calculated after cPD, since the first *Tr* drop (at any time, regardless of cPD) poorly correlated with PFS (Fig. S11).

In summary, after an early decline captured by cPD, residual T-DM1 efficacy is captured by patient-specific ctDNA trends (*Tr*).

### A ctDNA-guided algorithm for End of Treatment (cEoT)

Based on ctDNA waving and *Tr*, a branched decision algorithm is proposed that incorporates both cPD and post-cPD metrics to simulate/predict the timing of optimal, personalized ctDNA-guided End of Treatment (cEoT). In this algorithm, cPD does not directly trigger treatment decisions. Instead, it is suggested to prompt further intensified blood drawing, which in the GIM21 study was only available for patients seen at the PI site (w3, at every T-DM1 administration). During intensified blood drawing, evidence is sought (Fig. 5d) supporting cEoT. This is assigned at either (a) the second of two consecutive ctDNA increases, regardless of magnitude, or (b) the first ctDNA absolute increase after the first post-cPD *Tr* drop, whichever occurs first. If neither (a) nor (b) occurs, EoT coincides with PD (c), as per standard of care.

cEoT could be recorded in only 12/27 cPD-positive patients, mostly from the PI site (Fig. 5b). In this small patient subset, time to cEoT correlated with PFS (R^2^ = 0.86) (Fig. 5e). Interestingly, the regression coefficient was similarly high (R^2^ = 0.85) when the full algorithm (cEoT and EoT combined) was retrofitted to the entire set of 27 cPD-positive GIM21 patients (Fig. 5f). cEoT was recorded in 3/12 fast-progressors and 9/15 slow-progressors (PD before and after median PFS respectively; Fig. 5b). It may be calculated that the application of the full cEoT/EoT algorithm in place of cPD could have marginally delayed medical decision (42 days on average) in fast-progressors, but would have resulted in considerably extending treatment (256 additional days on average) in slow-progressors. Better alignment of cEoT with PFS may mitigate the risk to prematurely withdraw an effective treatment.

## Discussion

RECIST 1.1 was developed to assist in the routine serial monitoring of treatment efficacy. Conversion of continuous measurements (tumor size) into a simple set of discontinuous OR metrics (PD, SD, PR, CR) is crucial to support multiple binary (go/no-go) decision nodes at patient re-evaluation in advanced cancer. The minimal expectation is that cRECIST should be equally discontinuous, binary, intuitive, and robust. In addition, should cRECIST forecast outcome, particularly for individual patients, it would meet diverse clinical needs with a common, highly personalized tool. The present GIM21 study provides an initial framework to embed cRECIST into RECIST 1.1, and proposes novel metrics to overcome the inherent limitations of RECIST-like approaches when applied to outcome prediction.

Inspired by the previous LiqBreasTrack study, conducted in a similar setting [23], the GIM21 protocol was designed for staggered blood/CT scan collection and greater ctDNA data density at initial time points (w3/w9/w12 schedule; Fig. 1a). Unlike tumor-informed NGS/PCR approaches widely used to detect minimal residual disease [26], our bespoke NGS/dPCR assay captured and monitored genomic alterations exclusively in blood, not to miss adaptively acquired target ctDNAs arising de novo in approximately half of T-DM1-treated patients [23]. Based on Poisson distribution analysis in up to 20.000 nanoliter-size partitions, bespoke dPCR effectively normalized for wild-type alleles (VAF rather than copies/mL), and compensated for pre-analytical variation (sample processing, cfDNA recovery, purity, and concentration), eliminating the need for duplicate testing (Figs. 1, S1-S3). Robustness and low cost are key features for real-world implementation.

For comprehensive overview of all ctDNAs, VAF and CNVs were computed into a single unit measuring overall % ctDNA changes (Δ_ctDNA_; Fig. 1b). In addition, the RECIST 1.1 scoring system was mirrored as much as possible into cRECIST, each objective tumor response having a ctDNA counterpart: SD/cSD, PD/cPD, CR/cCR and PR/cPR. Comparison after OR/cOR mirroring highlighted a far more marked and dynamic response to T-DM1 treatment of ctDNA than tumor size, exemplified by the considerable phenotypic enrichment in cPD-positive GIM21 patients (50% approximately) experiencing SD as their best clinical response while undergoing a measurable (cPR/cCR) ctDNA drop (Fig. 3). In turn, OR/cOR divergence led to poor correlation between best cOR (deepest ctDNA drop) and outcome (PFS and OS, Fig. 4d and e). However, when ctDNA amounts were disregarded and a time-dependent variable was derived (cPFS, the time elapsed between T_0_ and cPD), correlations became apparent with best OR (Fig. 4c) and hence, although indirectly, with PFS (Fig. 4a). It is surprising that quantitative ctDNA changes, successfully applied by many groups to predict outcome (see for instance refs [10–20]) were instead inferior to cPFS (a time-dependent variable) in the GIM21 dataset (Fig. S9). Whereas the reasons for this specific discrepancy remain unclear at present, it is of note that cPFS implicitly incorporates a measure of ctDNA amounts and its changes. Whether a ‘hybrid’ computation of time and ctDNA amounts may help cRECIST implementation requires testing in different clinical settings and larger case collections.

Regardless, ctDNA amounts (in most previous studies) and/or time to ctDNA increase (cPFS in GIM21) appear to be accurate enough to classify broad patient subsets, but remain crude predictors of individual outcomes. This is unfortunate because personalized cEoT may have a strong clinical impact, as shown by the SERENA-6 study [27]. In this trial, breast cancer patients receiving an Aromatase Inhibitor (AI) in combination with a cycline depended kinase 4/6 inhibitor (CDK4/6i) were randomized to either continue this combination, or to be switched to a Specific Estrogen-Receptor Degrader (SERD) plus CDK4/6i on first evidence of ESR1 resistance mutations in blood. PFS improvement in the ctDNA-guided arm was striking (from 9.2 to 16.0 months). Simple computation and cEoT/therapeutic switch on first ctDNA increase make this achievement even more remarkable, but the SERENA-6 protocol may not unfortunately be generalized.

In SERENA-6, adaptive ESR1 mutations were detectable in a subset of 351 out of 3256 tested patients, and were the elective targets of a provenly superior class of drugs (SERDs) directly counteracting ESR1-driven resistance to treatment. Therapeutic switch was partial, since AI were replaced by SERD, but CDK4/6i was maintained. At variance with SERENA-6, in many settings (including the GIM21 setting) progression is driven by multiple heterogeneous cancer drivers, the available treatments may not mechanistically counteract resistance, and candidate anticancer drugs may belong to different classes and/or imply deep changes in therapeutic associations.

**Fig. 6:**
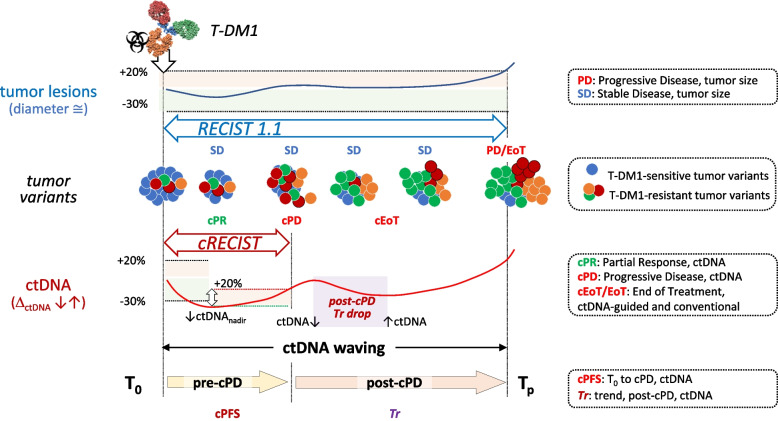
cRECIST and RECIST1.1: model, integration and metrics. An adaptive equilibrium is displayed between T-DM1 and tumor variants, in a hypothetical patient displaying a frequent phenotypic combination of best OR and best cOR, e.g. tumor stability combined with ctDNA response (SD/cPR; see Fig. 3c). Contraction and expansion of T-DM1-sensitive and T-DM1 resistant tumor variants are shown by blue and multicolor dots respectively, middle. The corresponding ORs and cORs are noted above and below on the two distinct RECIST 1.1 and cRECIST timelines. Double arrows define the applicable timeframes of ORs and cORs, e.g. from T_0_ to PD and T_0_ to cPD respectively. Tumor dynamics are poorly captured by RECIST 1.1 (‘flat’ blue trajectory and unchanged OR assignments), but recapitulated by successive Δ_ctDNA_ decreases and increases (‘waving’ red trajectory and changing cORs). ctDNA goes from baseline down to ctDNA nadir, up until and beyond cPD, and then down and up again post-cPD, a pattern termed ctDNA waving herein. The pre-cPD and post-cPD periods are monitored by two distinct, non-redundant ctDNA metrics: cPFS and *Tr*. cPFS is a cRECIST time-dependent variable based on two-point comparisons. It discriminates patient subsets with broadly different outcomes by measuring the time elapsed between T_0_ and cPD. *Tr* (three-point ctDNA *Tr*ends) is a non-cRECIST metric only applicable beyond the cRECIST timeframe. Combined with knowledge of cPFS, the duration of the first post-cPD *Tr* drop (boxed in red) is proposed to personalize ctDNA-assisted outcome prediction by incorporation into the cEoT decision algorithm

Possibly, SERENA-6 on the one hand, and many other published studies (including GIM21) on the other, highlight the best and worst cases of cEoT/therapeutic switch. In GIM21, cPD occurs early in most patients regardless of the depth and duration of clinical response. Afterwards, ctDNA undergoes successive expansion and contraction cycles, termed ctDNA waving, particularly evident in slow-progressors. To track ctDNA waving, cRECIST series of disconnected paired values had to be integrated with ctDNA trends (*Tr*) measured in consecutive overlapping triplets of time points (Fig. 6). Empirically, an ongoing *Tr* increase was associated with an immediate risk of progression. More interestingly, the duration of the first *Tr* drop, as long as occurring post-cPD (Fig. S11), was found to be informative, since it predicted individual outcomes much better than cPFS alone (R^2^ = 0.35 vs 0.86, Figs. 4h vs 5c). The simplest interpretation of ctDNA waving is that T-DM1 retains marginal efficacy during step-wise, multi-factorial acquisition of pharmacological resistance, and *Tr* reflects average tumor fitness throughout. Based on ctDNA waving and *Tr*, a branched cEoT decision algorithm is proposed that takes ctDNA waving into account, and aligns with the expected timing of PD much better than the earliest ctDNA increase, e.g. cPD (Fig. 5e/f, R^2^ = 0.85 to 0.86; compare with Fig. 4h, R^2^ = 0.35). Compared to cPD, the GIM21 algorithm would have marginally postponed (42 days on average) medical decision in fast progressors. However, it would have greatly mitigated the risk to discontinue an effective treatment too early in slow-progressors. It is expected that GIM21-like prediction algorithms and clinical decision trees might address medical situations more complex than those portrayed by SERENA-6. Exceptions notwithstanding, caution should be exercised before endorsing major ctDNA-guided deviations from a solid clinical standard such as RECIST 1.1.

A crucial, highly debated issue explored by GIM21 was that of ctDNA/cOR cut-offs. In the lack of extensive retrospective ctDNA data warehouses, the standard SD/PD (≥ 20%) and SD/PR (≤ 30%) cut-offs of RECIST 1.1 are supported by simplicity, immediacy, and mnemonic ease. To a first approximation they were therefore applied by default to GIM21 cRECIST (Fig. 1c). Remarkably, due to the considerable magnitude of most observed ctDNA changes, cOR and best cOR assignments remained largely cut-off-independent within the ≥ 20% to ≥ 50% range for cSD/cPD, and the ≥ 30% to ≥ 60% range for cSD/cCR, although at these expanded cut-offs outcome associations moderately worsened (Figs. 2b, S5-S8 and S10). GIM21 cut-offs bear analogies and differences with the expert opinion (based on literature review) of the LB-RECIST group. Also these authors noted that ctDNA prediction is largely cut-off independent, but suggested ≥ 10% and ≤ 50% changes as useful thresholds for ctDNA progression and response [8]. Alternative cSD cut-offs (≥ 10% cSD ≤ 10%) have been proposed in colorectal cancer [9]. Others have proposed that ctDNA changes are technically reliable as long as differences in dPCR measurements exceed confidence intervals [6]. Table 1 provides a concise overview of analogies, key points, and the underlying rationales for RECIST 1.1, LB-RECIST, and cRECIST. Altogether, the fine tuning of cSD cut-offs and post-cPD metrics appear to be major investigation goals. Prospective validation requires multi-arm randomized trials in different tumors and settings, and special validation schemes including those designed by the LB-RECIST group [8].

**Table 1:** Response evaluation criteria: a comparison

	***RECIST 1.1***	***LB-RECIST***	***cRECIST (herein)***
***Measurement***	tumor size	ctDNA levels	ctDNA levels
***Supporting evidence***	meta-analysis of huge data warehouses, multidisciplinary consensus, present standard of care	review of ctDNA literature	small prospective multicenter study, proof-of-concept
***Objective Criteria***	CR, PR, SD and PD	ctDNA increase and decrease	cCR, cPR, cSD and cPD, GIM21 algorithm/cEoT
***Cut-off: valid increase and decrease***	PR 30% ≤ SD ≥ 20% PD	ctDNA decrease ≤ 50% ctDNA increase ≥ 10% preferred*	mirroring RECIST 1.1 preferred
***Prognostic and predictive value***	increasing risk of progression: CR > PR > SD > PD	high or increasing ctDNA levels predict failure of checkpoint blockade and possibly targeted therapies	time variables (cPFS and cEoT) predict patient-specific outcomes; limited predictive value of ctDNA levels/changes
***Treatment decision tree***	binary (yes/no)	ctDNA levels as a continuous risk variable, but binary possible	binary
***End of Treatment***	on PD	on first ctDNA increase	on either cEoT or PD/EoT
***Application***	throughout disease course	same as RECIST 1.1	most valuable post-cPD

^*^ ≤ 10% SD ≥ 10% preferred in ref. 9

The model presented in Fig. 6 and described in the legend highlights OR/cOR divergence, integrates cRECIST into RECIST 1.1, and takes ctDNA waving into account. Complex trajectories may not be unique of ctDNA. Following adaptive HER2 polypeptide loss under T-DM1 therapeutic pressure, HER2 polypeptides were regained in tumor tissues and blood (sHER2) from a subset of GIM21 patients with a favorable outcome [28]. Late (e.g. post-cPD) residual responsiveness and waving tumor/drug equilibria may potentially apply to other targeted agents, tumors, settings, and companion analytes.

The GIM21 study design and protocols suffer from several limitations. These include a deliberately narrow focus on a single indication, low numerosity, and core-study inclusion of only 27 cRECIST-evaluable (cPD-positive) patients, although this selection did not apparently bias for outcome (Fig. 2c and d). In addition, it may retrospectively be argued that real-time testing would have missed new target ctDNAs appearing de novo at T_p_. These account for 18.1% of all ctDNAs (Fig. S1). Also, more intensive post-cPD blood drawing (as proposed in the GIM21 algorithm) could have been very useful to systematically measure the first post-cPD *Tr* drop and cEoT, that herein were measurable in 15 and 12 patients only, respectively. It is acknowledged that expanding cRECIST application beyond the present proof-of-concept will require intensive blood drawing and technical solutions capturing substantial numbers of target ctDNAs. Failure of ctDNA to detect intra-cranial progression may be particularly severe in GIM21, since brain metastases are frequent in HER2-positive breast cancer. This appears to be unavoidable, since spinal tap remains far superior to blood drawing [29], but possibly too invasive to be routinely applied. Finally, the GIM21 algorithm was inferred by retrofitting ctDNA measurements to the available GIM21 dataset, and was not prospectively tested.

Despite limitations, GIM21 is to our knowledge the first prospective study exploring the biological and clinical implications of cRECIST, providing initial evidence for irreducible conceptual differences between anatomical and ctDNA disease descriptions, and proposing practical computational approaches to personalize PD prediction.

## Conclusions

To conclude, cRECIST captures tumor dynamics differently from RECIST1.1. This may be challenging, but possibly offers opportunity for future integration. Three main practical cRECIST/cEoT implications may be foreseen: (a) in early drug development, cPFS and cEoT may represent early-on proxies of PFS and therapeutic efficacy; (b) in ctDNA-guided clinical trials, predefined cPFS and/or cEoT values may be prospectively applied to adaptively randomize the intention-to-treat population for either treatment maintenance or discontinuation/switch; and (c) in clinical practice, cPD and cEoT may flag individual patients for intensified post-cPD CT scan/ctDNA surveillance, and at the same time prevent premature withdrawal of an effective treatment in long-term responders displaying post-cPD ctDNA response/waving.

## Supplementary Information

Supplementary Material 1.

Supplementary Material 2.

## Data Availability

All raw data (total, and organized by figures) of ctDNA measurements are provided in an annotated spreadsheet as Table S2.

## Abbreviations

RECIST 1.1: Response Evaluation Criteria In Solid Tumors version 1.1
ctDNA: Circulating tumor DNA
cRECIST: CtDNA-RECIST
T-DM: Trastuzumab-emtansine
ORs and cORs: Objective Responses, clinical and ctDNA
PD: Progressive Disease
SD: Stable Disease
PR: Partial Response
CR: Complete Response
cPD: ctDNA-PD
cSD: ctDNA Stable Disease
cPR: ctDNA Partial Response
cCR: ctDNA Complete Response
NGS: Next Generation Sequencing
dPCR: Digital PCR
PET: Positron Emission Tomography
PERCIST: Response Criteria for Solid Tumors
iRECIST/imRECIST: Immune Response Criteria for Solid Tumors
GIM: Gruppo Italiano Mammella
ADC: Antibody–Drug Conjugate
IHC: ImmunoHistoChemistry
CT: Computerized Tomography
PFS: Progression-free Survival
OS: Overall Survival
cPFS: CtDNA-PFS
LT: Lead Time
