# Supplementary material for: Circulating tumor DNA and Response Evaluation Criteria In Solid Tumors: ctDNA-RECIST proof-of-concept in HER2-positive metastatic breast cancer
Source: J Exp Clin Cancer Res. 2026 Jan 20;45:67. doi: 10.1186/s13046-025-03605-2 (PMC12980920; doi:10.1186/s13046-025-03605-2)
Supplement: Supplementary file 1 — Supplementary Material 1. [file 13046_2025_3605_MOESM1_ESM.docx]

**Circulating tumor DNA and Response Evaluation Criteria In Solid Tumors: ctDNA-RECIST proof-of-concept in HER2-positive metastatic breast cancer**

Alessandra Fabi et al.

**SUPPLEMENTARY METHODS**

**Patient Population details**

Additional enrolment criteria were a left ventricular ejection fraction ≥50% on echocardiography or multiple-gated acquisition (MUGA; RRID:SCR_014072) scanning, an Eastern Cooperative Oncology Group performance status (ECOG-PS) of 0 or 1, a life expectancy >12 weeks, and adequate hematologic and end-organ function. Exclusion criteria included prior treatment with T-DM1; symptomatic central nervous system metastases, or treatment for these metastases within the last 2 months; history of symptomatic congestive heart failure or serious cardiac arrhythmia requiring treatment; history of myocardial infarction or unstable angina within the last 6 months; other prior malignancy, except for non-melanoma skin cancer and carcinoma in situ (of the cervix or bladder), unless diagnosed and/or successfully treated >5 years prior to accrual, and with no evidence of disease; any other comorbidity (including psychiatric illness) that could impair study participation or preclude informed consent; pregnant or lactating females; current or recent (i.e. in the last 4 weeks) participation in any interventional study.

**Toxicity management**

Dose delays, reductions, and discontinuation were as follows: the first dose reduction was to 3.0 mg/kg and the second one to 2.4 mg/kg (dose escalation was not allowed after a dose reduction). If a toxic event did not resolve to grade 1 level or baseline status within 42 days after the most recent dose, treatment was discontinued.

**cfDNA pre-analytical processing**

Blood was prospectively collected in K_2_EDTA vacutainers, processed within 30 min by the so-called 2-spin protocol, and stored in single-use aliquots at -80°C until shipment (in dry ice) to the GIM21 central lab for testing. Circulating cell-free DNA (cfDNA) was purified by the QIAmp ctDNA kit (Qiagen), quantitated with Qubit (RRID:SCR_020553), and retrospectively assessed by Next Generation Sequencing (NGS) Oncomine Pan-Cancer and QuantStudio 3D digital PCR (dPCR), both from ThermoFisher Scientific.

**SUPPLEMENTARY RESULTS**

**Circulating genomic alterations**

Targeted NGS at T_0_ and/or T_6_ and/or T_p_ from the entire dataset of 38 ctDNA-positive patients revealed circulating genomic alterations (n=54, of which 47 SNVs and 7 CNVs from 16 distinct genes) in 496 blood drawings. Alterations were 1 to 5 (median 2) per patient (Fig. S1a). Some alterations recurred in more than one patient, resulting in longitudinal monitoring of 78 mutational hits, e.g. 78 target ctDNAs altogether. ERBB2 was the only gene displaying both SNVs and CNVs. All 47 SNVs and 3 CNVs (HER2, MYC, and FGFR1) were monitored by bespoke digital PCR (dPCR), whereas NGS remained the sole testing method for the remaining 4 CNVs (CCND1, CCND2, CDK4, and FGFR3). Of 78 target ctDNAs, 72 were present at either or both T_0_ and T_p_, and were distributed as follows: 15 (20.8%) at T_0_ only, 13 (18.1%) at T_p_ only, and 44 (61.1%) at both T_0_ and T_p_ (Fig. S1b and c). Alterations exclusively detectable at T_p_ were tested retrospectively, and would have been missed by real-time prospective testing. Raw ctDNA measurements are provided in an annotated spreadsheet as Table S2. A single SNV (not shown in the Oncoprint display of genomic alterations in Fig. S1a) was associated with Clonal Hematopoiesis of indetermined Potential (CHiP), as confirmed by dPCR testing of genomic DNA from white blood cells.


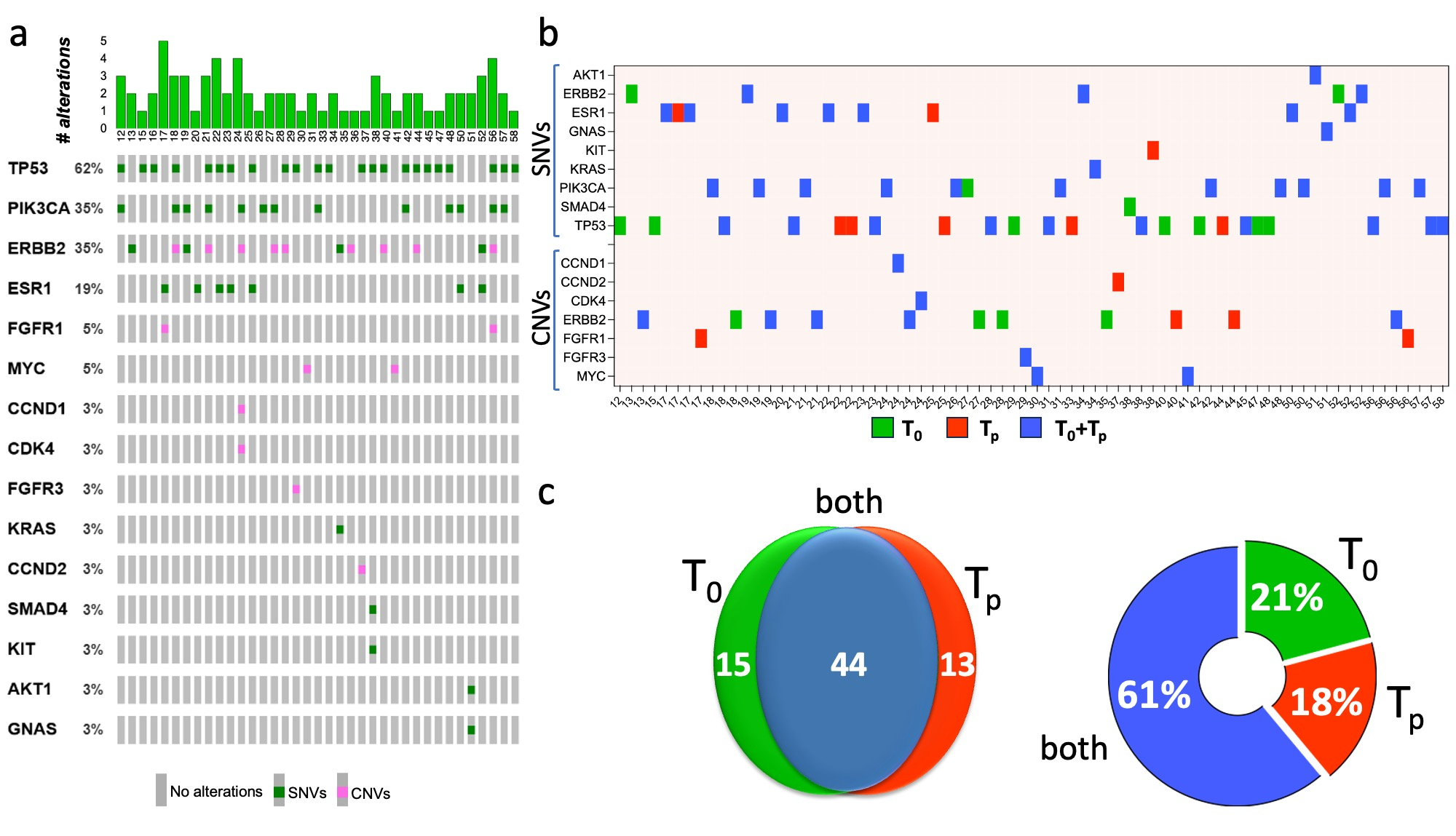


**Fig. S1. Circulating genomic alterations during T-DM1 treatment.** (a) Graphical display of genomic alterations (Oncoprint) detected in blood from the 38 ctDNA-positive GIM21 patients. The number and gene distribution of genomic alterations (SNVs and CNVs) are shown for each patient (numbered on top). (b) SNVs and CNVs detectable at baseline (T_0_), progression (T_p_), or both, listed by gene. (c) Venn diagram and donut chart of the mutational hits detected at either or both T_0_ and T_p_.

From 56% to 85% of all mutation hits (depending on type and timing) were actionable (Chakravarty D, et al. JCO Precis Oncol 2017) at OncoKB ([https://www.oncokb.org](https://www.oncokb.org/)) level 1 (Fig. S2a and b). Due to opposing trends of different ctDNA species, overall ctDNA abundance (both SNVs and CNVs) was conserved between T_0_ and T_p_ (Fig. S2b). Altogether, these results demonstrate extensive dynamic changes in oncogenic drivers during treatment.


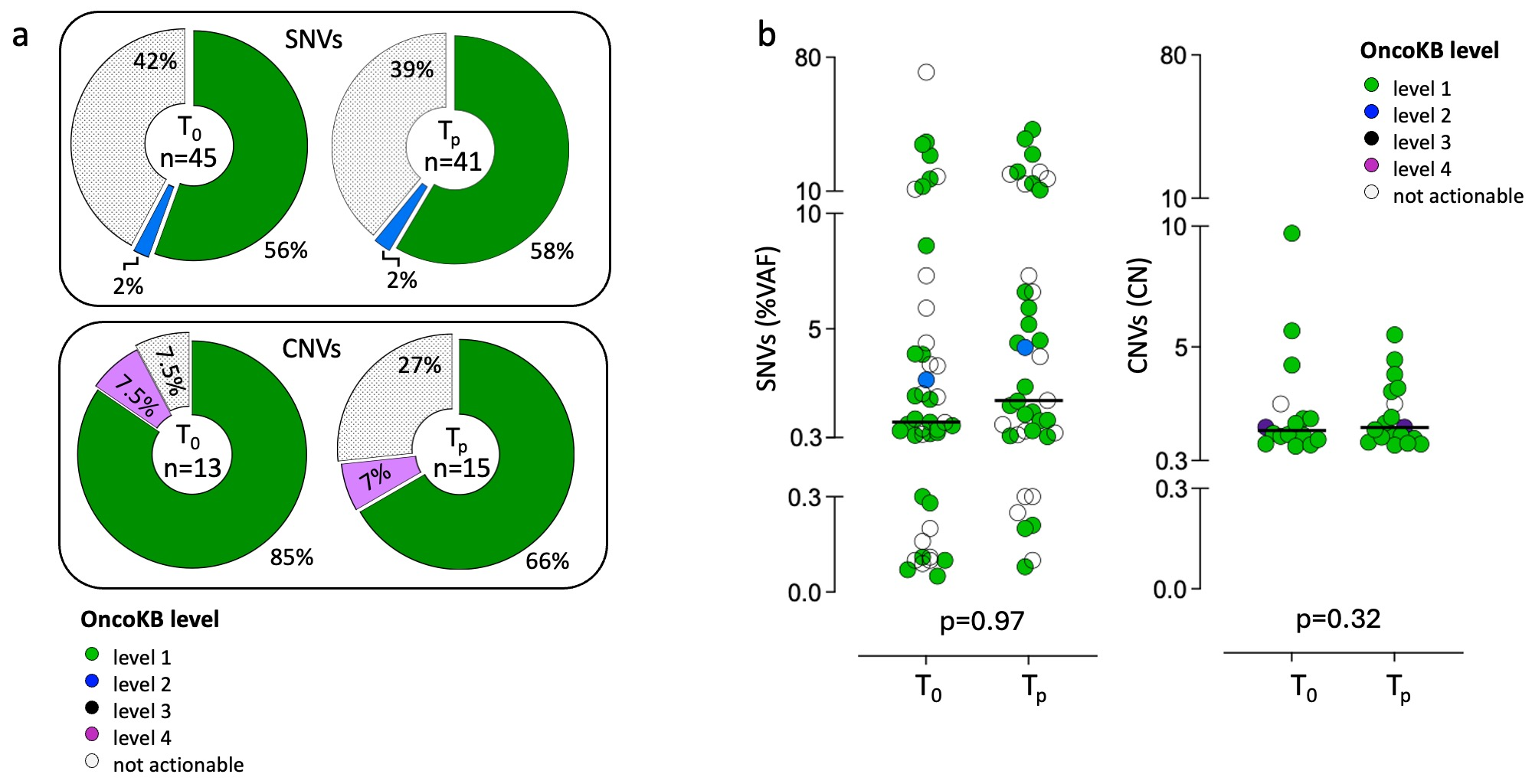


**Fig. S2. Actionable genomic alterations during T-DM1 treatment.** SNVs and CNVs in the blood of 38 ctDNA-positive patients classified by: (a) Donut chart of OncoKB level; and (b) dot plots of OncoKB level plus abundance (VAF and copy numbers), separately calculated at T_0_ and T_p_. Differences at *t* test non-significant.

**Technical validation of ctDNA testing**

To exclude trivial errors, technical validation was two-fold: (a) orthogonal NGS/dPCR testing, and (b) dPCR re-testing. Orthogonal testing was carried out on blood obtained at T_0_. All SNVs called by NGS were confirmed by dPCR (47/47, 100%). Variant Allele Frequency (VAF) was remarkably concordant in 32 paired determinations, as shown by regression analysis (Fig. S3a, top: R^2^=0.98; 95% CI 0.95 to 1.06; beta coefficient 1.006), highlighting reproducibility

**
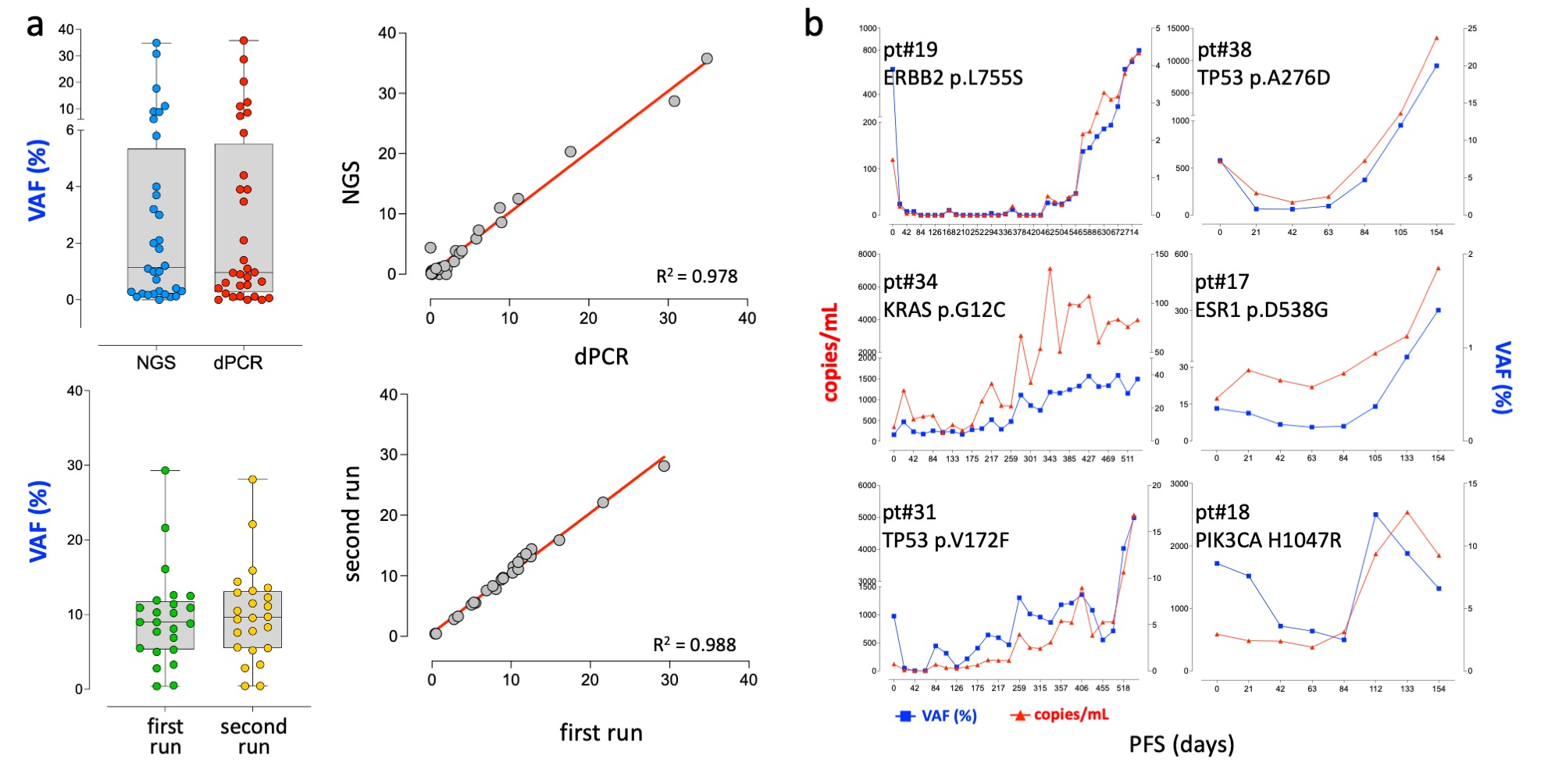
**

**Fig. S3. dPCR accuracy, reproducibility, repeatability and preferred metric.** (a) Top left: scatter plot of VAF values (raw data) detected by NGS/dPCR orthogonal testing of 32 representative cfDNAs collected at T_0_. Next: regression analysis of the same data after NGS/dPCR pairing. Bottom left: scatter plot of VAF values (raw data) detected by repeated (first run vs second run) dPCR testing of 24 cfDNAs. Next: regression analysis of the same data (paired VAF values). (b) Metric comparison: ctDNA trajectories (VAF vs copies/mL) of serial dPCR testing from 6 representative patients.

in ctDNA measurements between different assays. dPCR repeatability was assessed by testing twice a randomly selected subset of 23 cfDNA samples. After the first testing round, cfDNAs were frozen again at -80°C, thawed 730 to 1278 days (median 1003 days) later, and re-tested using distinct aliquots of the same custom-designed primers and assays, and the same dPCR equipment, but with newly prepared dPCR amplification mixes.

Also in this case VAF values were essentially superimposable (R^2^=0.99; 95% CI 0.95 to 1.04; beta coefficient 0.991; Fig. S3a, bottom). Thus, inter-assay (NGS vs dPCR) and intra-assay (dPCR repeats) testing results were in excellent agreement for both SNVs and CNVs (not shown), showing that dPCR was accurate, reproducible, and repeatable enough to make duplicate testing unnecessary. dPCR repeatability, supported by robust digital microchip reading of up to 20.000 replicate nanowells and Poisson distribution analysis, as well as moderate cost, led to dPCR selection for systematic testing.

**Identification of VAF and CN as the optimal cRECIST data inputs**

SNVs may be measured by either copies/mL or Variant allele frequency (VAF), e.g. in absolute and relative (to the WT allele) units, respectively. In a subset of 12 patients, recruited at the Principal Investigator’s site, ctDNA testing was intensive, in that it was carried out at each T-DM1 cycle even after T_3_ (w3 schedule instead of w3/w9), resulting in a more detailed ctDNA timeline. This data-dense subset was selected to compare ctDNA trajectories. Plotting copies/mL vs VAF revealed similar trajectories, but in at least some cases plots were less variable for VAF than copies/mL (representative results in Fig. S3b). Due to smoother ctDNA trajectories, VAF was selected as the primary data source for cRECIST scoring.

**Divergent trajectories: target lesions vs target ctDNAs**

For a detailed longitudinal point-by-point (T_0_ to T_p_) evaluation of the individual trajectories of all 113 tumor lesions and 78 target ctDNAs, lesion diameters and Δ_ctDNA_ values were elaborated as follows. Default RECIST 1.1 upper and lower cut-offs discriminating SD from PD and CR respectively (≥20% and ≤30%, see Fig. 1) were applied to discriminate cSD from cPD and cCR. Changes in size/levels of each tumor lesion and ctDNA species were scored between consecutive time points (T_n_ vs T_n-1_). For consistency between tumor and ctDNA, only default cut-offs were applied. One of 5 possible responses was noted at each time point, e.g. increase, decrease, no change, gain (de novo appearance), and loss (disappearance). When a tumor lesion or ctDNA was undetectable, this was specifically noted. Responses were color-coded consistently between lesions and ctDNAs, and displayed as timelines and donut charts. Timelines revealed that, compared to lesion diameter, changes in Δ_ctDNA_ values were more numerous, frequent, discordant, asynchronous and extreme, including occasional direct switch from loss to gain at immediately consecutive time points (Fig. S4a). For instance, in pt#52 progression (PD) was due to the *de novo* appearance of a novel lung metastasis. Meanwhile, three different ctDNAs had undergone strikingly discordant trajectories. Likewise, pt#17 displayed 5 index lesions: 3 remained stable or partially responded, and 2 were new lesions determining PD. In contrast, all 4 ctDNAs diverged, and one of them underwent two consecutive changes in OR. As to pt#40, a dimensional increase was noted of only 1 of 5 metastases, whereas both detectable ctDNAs displayed opposite and multi-segmented (waving) trajectories. Similar discordant trajectories were particularly evident in pt#16 and pt#42. Altogether, application of the above criteria classified objective responses into 18 different patterns (5 of which simple and 13 complex, all listed in the legend) defining tumor and ctDNA changes. Relative frequencies of response patterns are displayed as donut charts (Fig. S4b). Presence of more than one change in a timeline was observed in only 33% of tumor lesions, but in 69% of ctDNAs. Viceversa, stability (no change exceeding cut-offs) was seen in 35% of tumor lesions but 8% only of ctDNAs.


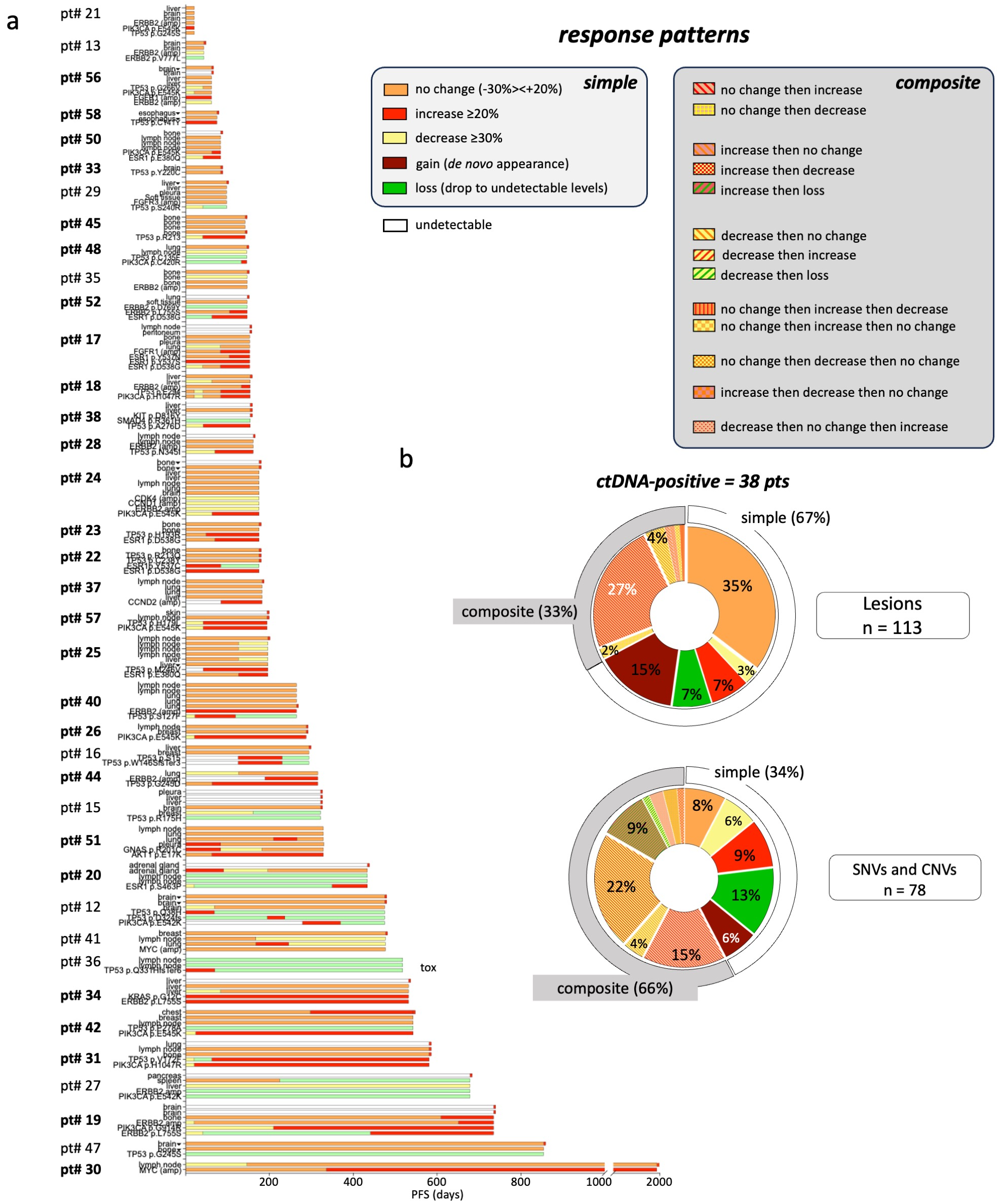


**Fig. S4. Response patterns of circulating genomic alterations during T-DM1 treatment.** (a) Paired timelines (individual tumor lesions, top; individual ctDNAs, bottom) displaying in succession one of five possible responses (color-coded and defined in the top left box) in each of the 38 ctDNA-positive patients. When undetectable, tumor lesions and ctDNAs were depicted as open bars. Identical response cutoffs (≥20%; ≤30% and undetectable) were applied to score tumor lesions and ctDNAs. Responses at any given time point were calculated relative to the time point immediately before (T_n_ vs T_n-1_). All 38 ctDNA-positives are shown; the 27 cPD-positive patients are boldface. Non-target tumor lesions are marked by a triangle. pt#36 discontinued T-DM1 due to toxicity (tox) while in complete response (both clinical and ctDNA). (b) Donut charts enumerating simple and composite response patterns. Simple and composite patterns (defined in top grey boxes) are coded by solid and stippled colors, the latter generated by combining the colors of the simple responses contributing to the observed composite pattern.

Donut charts also provide synoptic evidence for roughly reciprocal abundance of simple and composite (more than one change in the timeline) response patterns in tumor lesions (67% and 33%) vs ctDNAs (34% and 66%).

In summary, evolutionary divergence of ctDNA variants largely exceeded dissociated radiological responses among tumor lesions measured by CT scans. Conversely, stable disease was rare from the ctDNA standpoint.

**Exploring a range of cSD/cPD and cSD/cPR scoring cut-offs: impact on cRECIST timelines, and cOR/best cOR assignments.**

Cut-offs between ≥20% and ≥50% for cSD/cPD, and between ≤30% and ≤60% for cSD/cPR minimally affected the timelines, pie charts and Pearson’s matrices of Fig. 3 (compare Fig. S5 to Figs. S6-S8). Cut-offs had to be raised to ≥100%/≤100% to observe similar OR and cOR profiles resulting from drastic quenching of ctDNA progression/response, and hence increased cSD representation in the dataset. At these cut-offs cPD was completely missed in three patients, delayed slightly in one (pt 38) and delayed considerably (coincident with PD) in two (pts. 50 and 40). This questions the added value of ctDNA testing at such extreme cut-offs.


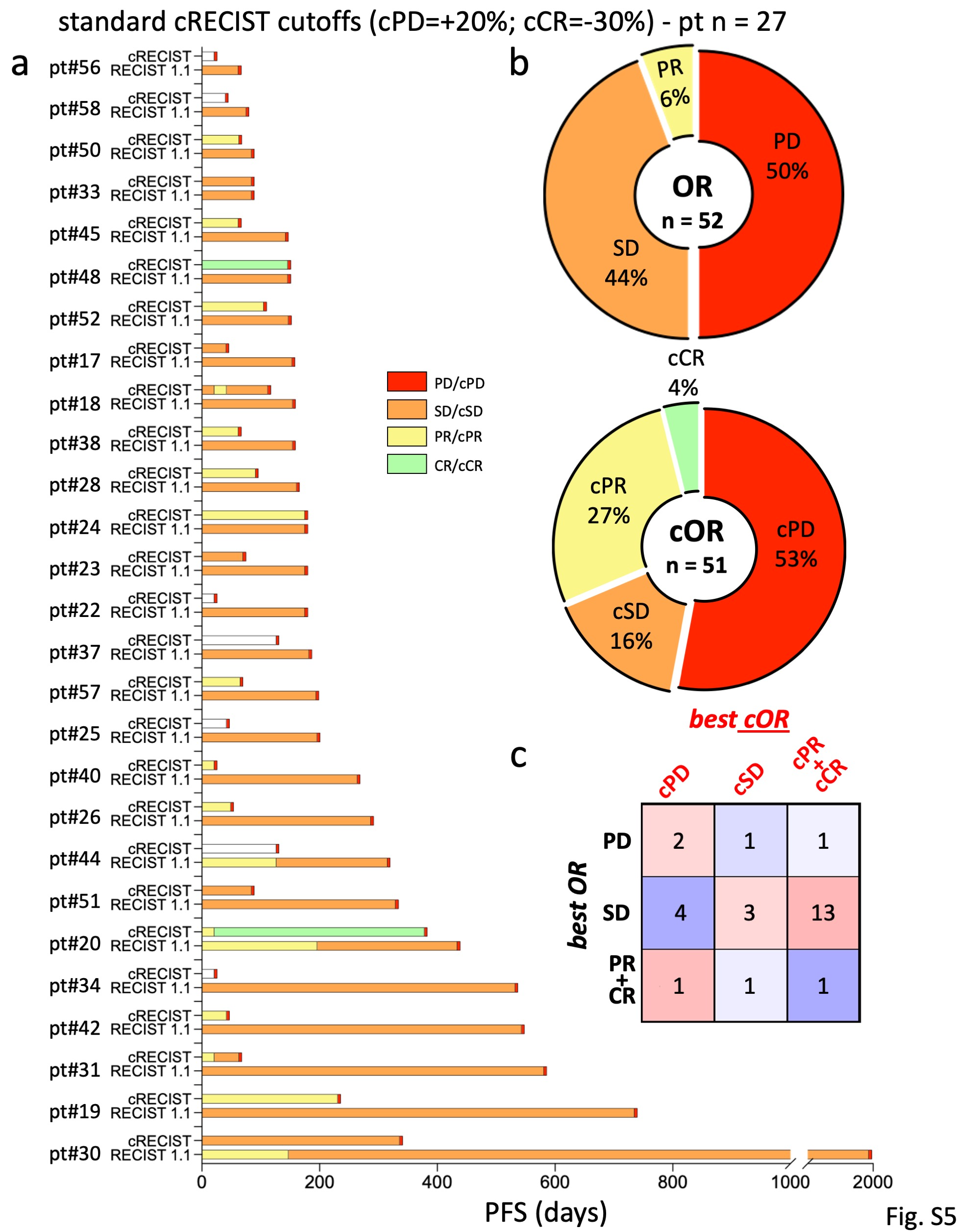


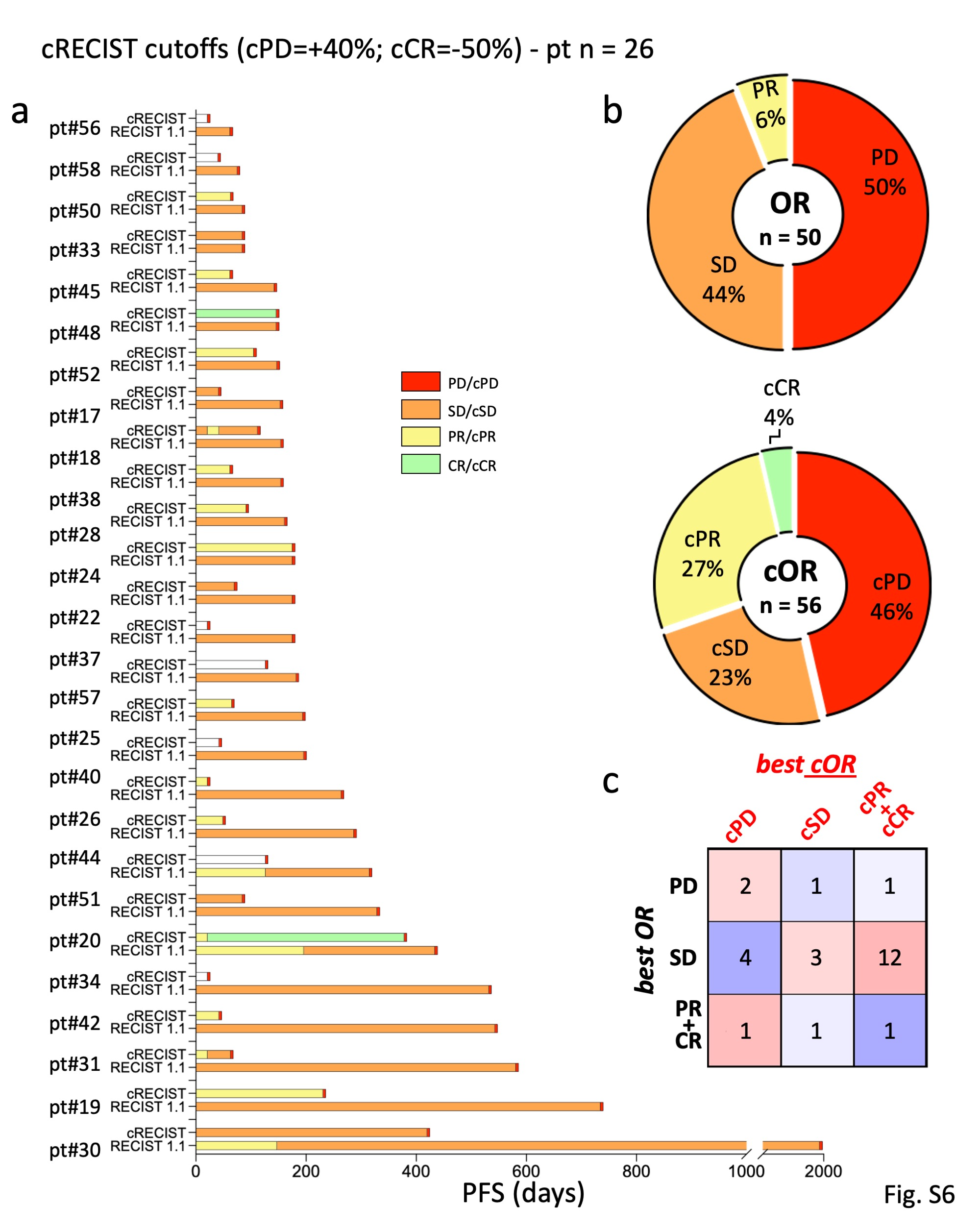


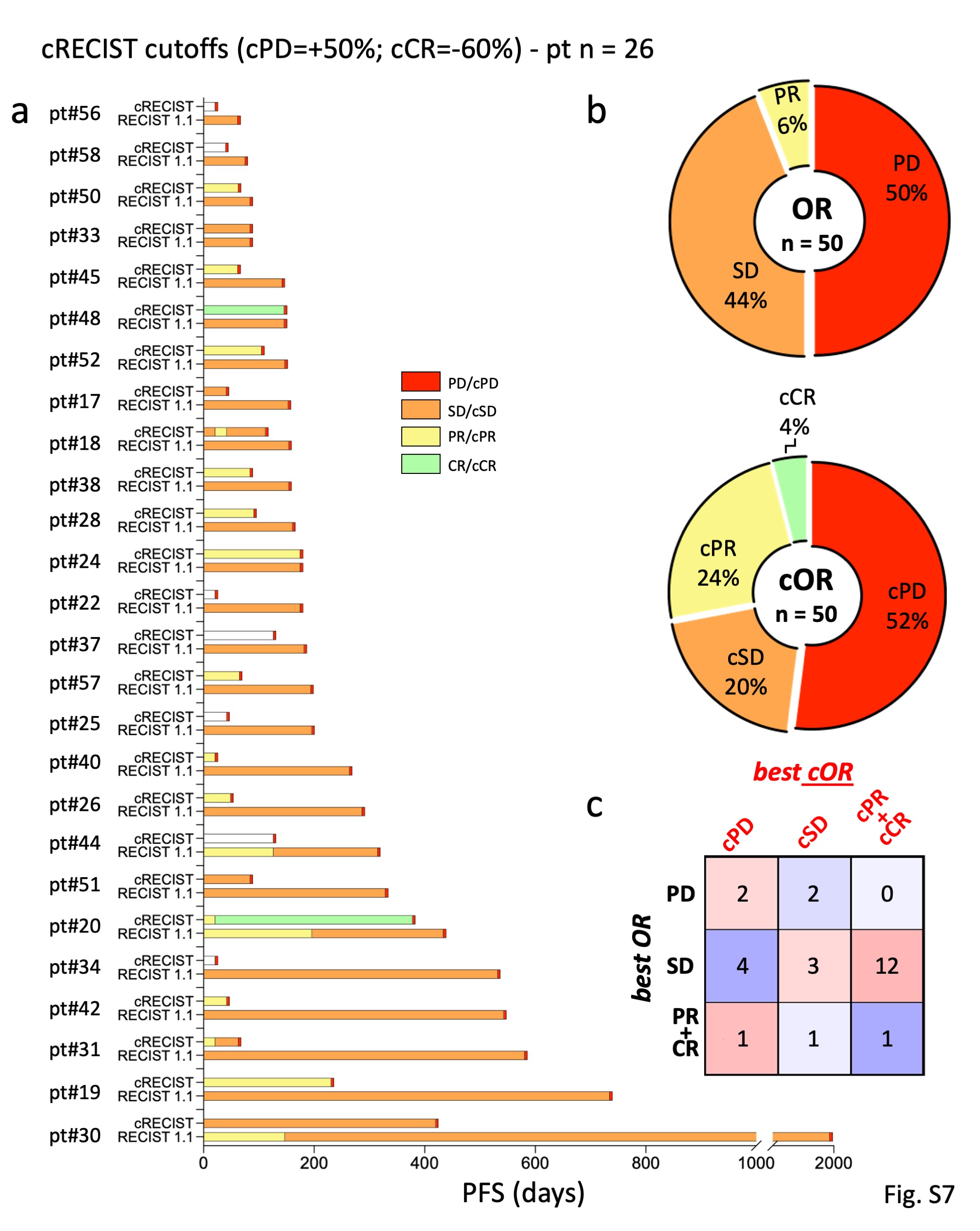


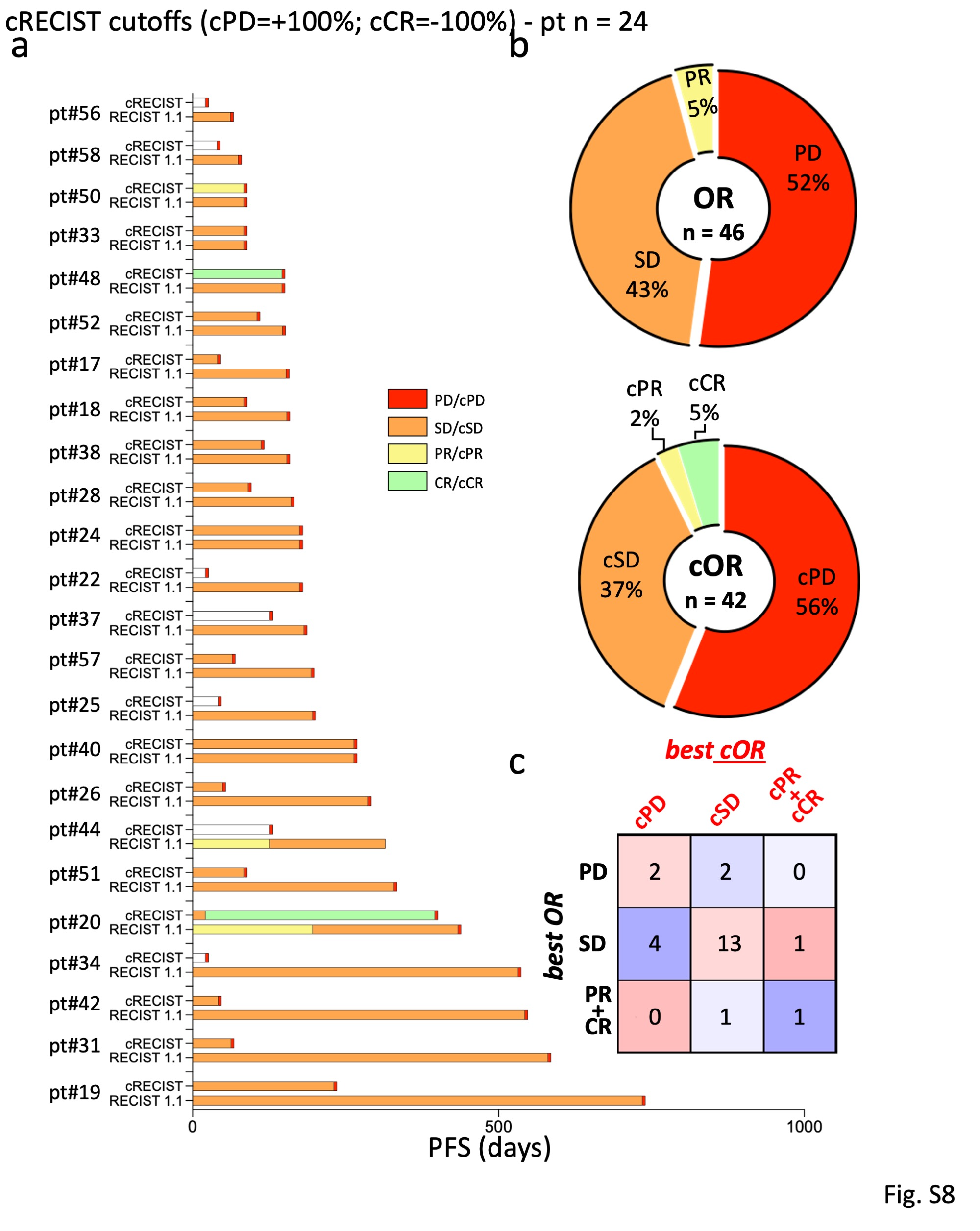


**Fig. S5-S8. ctDNA amounts and outcome.** Effect of the stringency of cPD/cCR scoring criteria on the timelines, pie charts and Pearson’s correlation matrix presented in Fig. 3 (see legend). Results with default cut-offs are presented first (Fig. S5 is a replicate of Fig. 3), and then 3 simulations (S6-S8) are provided at the indicated, progressively more stringent upper and lower cSD cut-offs.

**ctDNA amounts and outcome**

Correlations with outcome were explored by comparing patients differing in absolute ctDNA levels and their dynamic changes. These were measured by diverse, continuous and discontinuous (cRECIST) variables. Discontinuous cRECIST variables were assessed by applying both default and increasingly stringent cut-offs discriminating cSD from cPD and cPR. The following correlations were investigated (Fig. S9): (a and b) PFS in patients with ctDNA amounts (SNVs + CNVs) below study mean vs patients with ctDNA amounts above study mean; PFS in patients with ctDNA amounts (SNVs only) below study mean vs patients with ctDNA amounts above study mean (not shown); (c) PFS in patients with increases in ctDNA trends (T_p_>T_0_) vs patients with decreases (T_0_>T_p_); (d and e) PFS and OS in patients with an early objective ctDNA response at T_1_ vs patients with no response (PFS and OS were also calculated in patients with an early objective ctDNA response at T_2_ and T_3,_ but these were similarly non-significant and are not shown); (f-i) PFS and OS in patients with early objective ctDNA response at T_1_ (as above) vs no response, different cSD cut-offs; (j) patients with complete early (at T_1_) clearance of at least one target ctDNA vs patients with no clearance; (k and l) PFS and LT by time to first increase (any individual ctDNA species); (m, n, o) PFS by early changes in SNV abundance at T_1_, T_2_ and T_3_ vs T_0_, calculated by a continuous ΔVAF scale (ratios). In summary, absolute ctDNA levels, the entity of their increase/decrease, and early kinetics did not correlate with the outcomes of either patient subsets or individual patients regardless of cSD cut-offs.


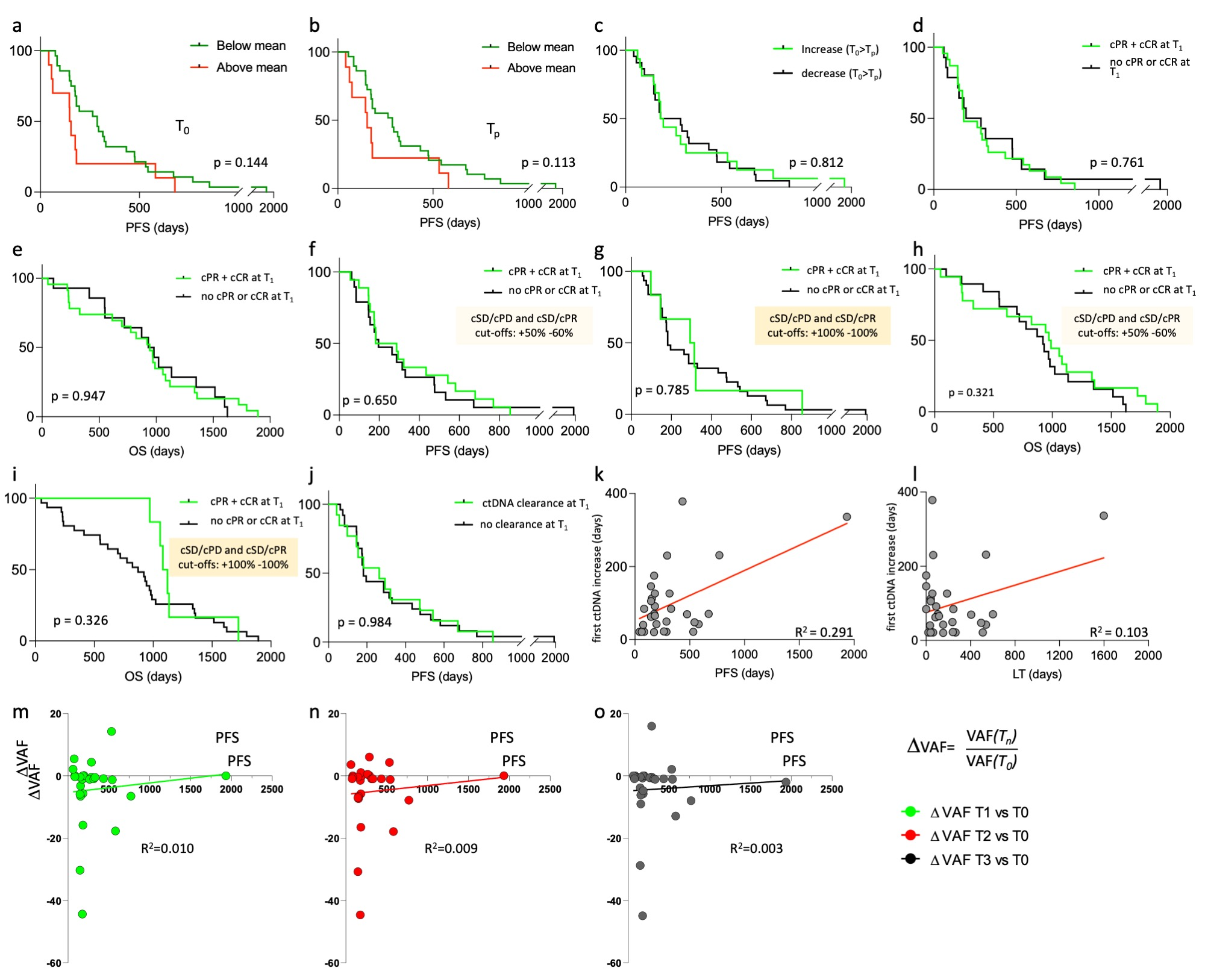


**Fig. S9. ctDNA amounts and outcome.** Kaplan-Meier and regression plots: outcome comparisons (PFS and/or OS as indicated) between patients differing in: (a) absolute ctDNA levels (SNVs and CNVs altogether) calculated at T_0_ as follows: Σ[VAF*(T_0_)* + CN*(T_0_*)], below mean vs above mean. (b) ctDNA levels calculated as above but at T_p_: Σ[VAF*(T_p_)* + CN*(T_p_*)], below mean vs above mean. (c) ctDNA increase vs decrease (means calculated as above), T_0_ vs T_p_. (d) Achievement of an objective response at T_1_: responders vs non-responders, PFS. (e) As above, OS. (f; g) As above, PFS, different cut-offs. (h, i) As above, OS, different cut-offs. (j) Complete clearance of at least one target ctDNA at T_1_ vs all others. (k) Time elapsed (T_0_ to the time of the first recorded ≥20%increase in any individual ctDNA species), plotted vs PFS. (l) Same as above, plotted vs Lead Time (LT). (m) ΔVAF (T_1_/T_0_) vs PFS. (n) ΔVAF=T_2_/T_0_ vs PFS. (o) ΔVAF=T_3_/T_0_ vs PFS. ΔVAF is the ratio of average VAF values (all detectable SNVs). Negative ΔVAF values are possible when ctDNA decreases.

**Impact of cPD/cCR scoring criteria on GIM21 metrics and outcome**

Different cSD upper and lower cut-offs, particularly when within the ≥50% to ≤60% range, had minimal effect on the number of patients scored as cPD-positive, as well as cPFS and lead time (LT), as shown by dot plot comparison (Fig. S10a and b). These changes worsened best OR/PFS correlation (compare Fig. S10c and d with Kaplan Meyer analysis in Fig. 4c), but had limited if any effect on linear regression plots between LT and PFS (Fig. S10e and h compared to Fig. 4j). In summary, correlations pointing to the post-cPD period (LT/PFS overlap) appear to be resilient to cut-off changes. Therefore, ctDNA variables measured in this period are likely to have the strongest impact on outcome in the GIM21 setting.


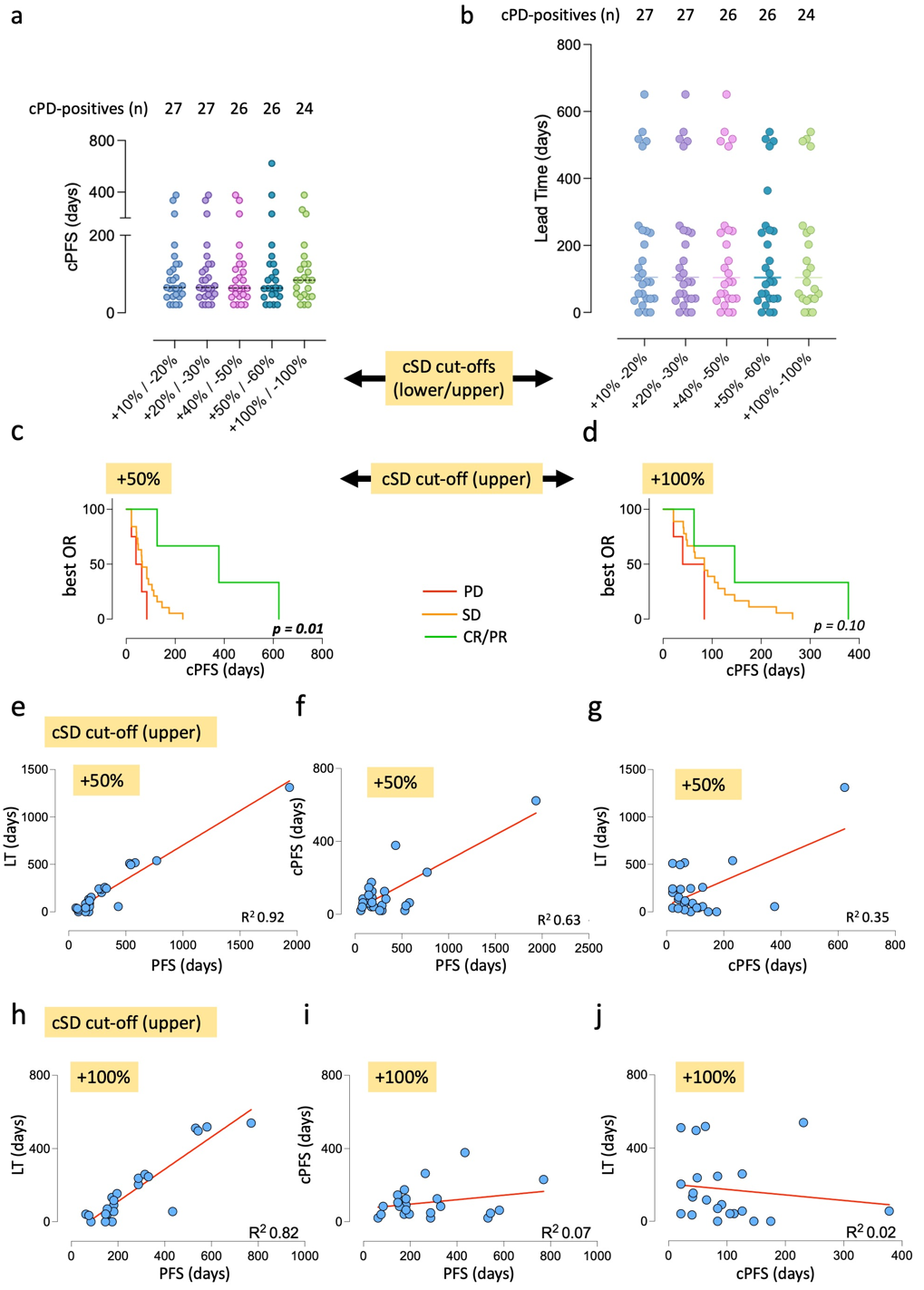


**Fig. S10. cRECIST cut-offs and outcome.** The impact of scalar deviations from the default upper and lower cSD cut-offs is evaluated on the following variables: (a, b) the number of patients with cPD, and the duration of cPFS and lead time (LT) visualized by dot plots: means of cPFS and LT are displayed; (c, d) correlation between best OR and cPFS (same as Fig. 4c); (e-j) linear regression plots between any two of LT, PFS and cPFS. For definition of the variables see main text.

***Only post-cPD Tr* correlates with outcome**

When the first Tr drop was considered, regardless of its timing (whether before or after cPD), correlation with PFS was poor (Fig. S11 compared to Fig. 5c).

**Fig. S11. Tr and outcome**

Regression analysis: first observed *Tr* drop (regardless of cPD) vs PFS.


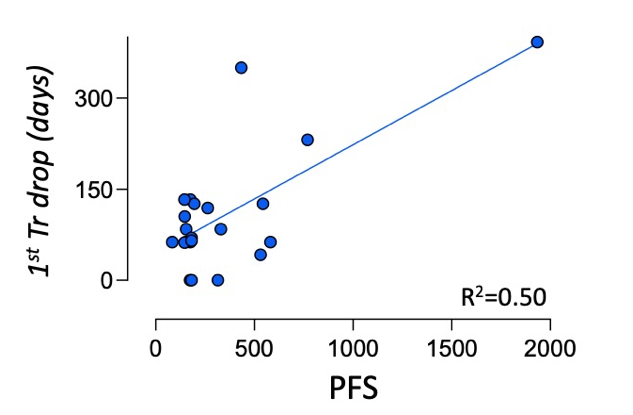


Table S1

**Demographics and clinical pathological features of GIM21 patients**

| ***cPD-positive patients (n=27)*** |  |
| --- | --- |
|  |  |
| **Age, years (range)** | 57.8 (33-79) |
|  |  |
| **Previous lines of therapy for metastatic disease*** |  |
| 0 | 0 |
| 1 | 27 |
|  |  |
| Trastuzumab | 2 |
| Trastuzumab + Chemotherapy | 3 |
| Trastuzumab + Pertuzumab | 3 |
| Trastuzumab + Pertuzumab + Chemotherapy | 18 |
|  |  |
| **Dominant Metastatic sites** |  |
| Lymph Node | 17 |
| Bone | 14 |
| Lung | 11 |
| Liver | 8 |
| Brain | 4 |
| Pleura | 4 |
| Breast | 2 |
| Soft tissues | 2 |
|  |  |
| **Number of metastatic sites per patient** |  |
| 1 | 5 |
| 2 | 12 |
| > 3 | 10 |

| ***cPD-negative patients (n=16)*** |  |
| --- | --- |
|  |  |
| **Age, years (range)** | 56.3 (41-82) |
|  |  |
| **Previous lines of therapy for metastatic disease*** |  |
| 1 | 16 |
|  |  |
| Trastuzumab | 2 |
| Trastuzumab + Chemotherapy | 3 |
| Trastuzumab + Pertuzumab | 2 |
| Trastuzumab + Pertuzumab + Chemotherapy | 8 |
|  |  |
| **Dominant Metastatic sites** |  |
| Brain | 7 |
| Bone | 6 |
| Liver | 6 |
| Breast | 5 |
| Lung | 4 |
| Lymph node | 4 |
| Pleura | 3 |
| Soft tissues | 2 |
|  |  |
| **Number of metastatic sites per patient** |  |
| 1 | 6 |
| 2 | 3 |
| > 3 | 7 |

*Previous lines: Lapatinib plus Capecitabine, Trastuzumab plus Vinorelbine, Trastuzumab plus Carboplatin.
